# Three Prognostic Biomarkers Correlate with Immune Checkpoint Blockade Response in Bladder Urothelial Carcinoma

**DOI:** 10.1155/2022/3342666

**Published:** 2022-05-26

**Authors:** Ya Guo, Yin Bin Zhang, Yi Li, Wang Hui Su, Shan He, Shu Pei Pan, Kun Xu, Wei Hua Kou

**Affiliations:** ^1^Department of Radiation Oncology, The Second Affiliated Hospital of Medical College, Xi'an Jiao Tong University, Xi'an, 710004 Shaanxi, China; ^2^Department of Oncology, The Second Affiliated Hospital of Medical College, Xi'an Jiao Tong University, Xi'an, 710004 Shaanxi, China; ^3^Department of Stomatology, The Second Affiliated Hospital of Medical College, Xi'an Jiao Tong University, Xi'an, 710004 Shaanxi, China

## Abstract

**Aim:**

We aim to develop a signature that could accurately predict prognosis and evaluate the response to immune checkpoint blockade (ICB) in bladder urothelial carcinoma (BLCA).

**Methods:**

Based on comprehensive analysis of public database, we identified prognosis-related hub genes and investigated their predictive values for the ICB response in BLCA.

**Results:**

Among 69 common DEGs, three genes (AURKA, BIRC5, and CKS1B) were associated with poor prognosis, and which were related to histological subtypes, TP53 mutation status, and the C2 (IFN-gamma dominant) subtype. Three genes and their related risk model can effectively predict the response of immunotherapy. Their related drugs were identified through analysis of drug bank database.

**Conclusions:**

Three genes could predict prognosis and evaluate the response to ICB in BLCA.

## 1. Background

Bladder cancer is the ninth most common malignant tumor worldwide, and bladder urothelial carcinoma (BLCA) is its most frequent pathological type [1]. Despite the development of diagnostic and treatment techniques, the 5-year survival rate of patients with BLCA varies from 5% to 70%, and their prognosis remains unfavorable [2]. Detection of biomarkers can indicate a particular disease state and can be used in screening for differential prognosis, evaluation of treatment response, and monitoring of disease progression [3]. As demonstrated in different studies, the availability of several cancer databases and a comprehensive bioinformatics analysis has allowed for the accurate identification of key biomarkers for early diagnosis of malignancies or prediction of prognosis and cancer recurrence [4–7]. Therefore, it is reliable to develop useful biomarkers for predicting BLCA prognosis based on systematic bioinformatics analysis.

BLCA is the 10th most common malignant tumor worldwide, which is divided into nonmuscle-invasive BLCA (NMIBC) and muscle-invasive (MIBC). MIBC has an unfavorable prognosis with a 5-year overall survival (OS) of approximately 50% [8, 9]. In recent years, cancer immunotherapies by immune checkpoint blockade (ICB) have attracted considerable attention for its influence on the treatment of locally advanced and metastatic BLCA [10]. Recent studies reported that immune checkpoint blockade (ICB) has promising improved survival rates in individuals with advanced BLCA with a high tumor mutation burden (TMB) and neoantigens. Patients with tumors that overexpression of programmed cell death 1 receptor (PD-1) or its ligand (PD-L1) appear to benefit most from this therapy [9, 11]. However, only a proportion of patients respond to treatment with ICB, the results of immunotherapy are still not satisfactory [12, 13]. Therefore, immunotherapy needs to continue to improve in terms of improvement in their effectiveness in the treatment of BLCA.

Previous studies have reported that highly infiltrating lymphocytes are related to the prognosis of BLCA [14]. The evidence indicates that CD8+ T cells are involved in tumor adaptive immunity, and their infiltration is associated with prognosis [15, 16]. Programmed death ligand 1 (PD-L1), also known as B7-H1 or CD274, is involved in inhibiting T cell-mediated antitumor immunity through interaction with PD-1 [17, 18]. Previous studies have reported that high expression of PD-L1 is associated with worse cancer outcomes in various malignancies [19]. CD8+ T cell infiltration and tumor mutation burden (TMB) have been reported to be correlated with response to atezolizumab (anti-PD-L1) in metastatic urothelial cancer (mUC) [20–22]. However, none of these factors is sufficient to achieve accurate outcome prediction, and identification of ICB response biomarkers is a critical challenge in the field [23]. In general, there is an urgent need to identify key biomarkers that can be used to effectively evaluate the response to immunotherapy in BLCA patients.

In this study, we first identified DEGs from the GEPIA (Gene Expression Profiling Interactive Analysis) and Oncomine databases and then identified the overlapping DEGs between them using a Venn diagram. We further performed gene enrichment and protein–protein interaction (PPI) analyses to select hub genes. Moreover, prognosis-related hub genes were identified using comprehensive bioinformatics analysis and confirmed in three online databases. We then explored the expression of the key prognosis-related genes with different clinical factors using the UALCAN and TISIDB database. Finally, we evaluated whether the risk model based on three key genes could be used to predict immunotherapy response in BLCA.

## 2. Methods

### 2.1. Identification of DEGs

GEPIA (http://gepia.cancer-pku.cn/) is an online network tool based on data from The Cancer Genome Atlas (TCGA) and GTEx, which can be used to study interactions between DEGs, as well as for survival analysis, profile plotting, and detection of similar genes. Oncomine is an online resource containing microarray data (https://www.Oncomine.org) [24]. In this study, we first used the GEPIA and Oncomine databases to identify DEGs by comparison of tumor samples with normal samples. RNA-Seq data from the TCGA-based (The Cancer Genome Atlas) GEPIA database was performed to identify differentially expressed genes (DEGs) between 404 tumor and 19 normal tissues. ANOVA method was used to obtain the differently expressed genes (DEGs), mRNAs with *q* < 0.01 and |log2 fold change (FC)| ≥ 2 were selected as DEGs. In the Oncomine database, 288 samples from TCGA gene expression dataset were used to screen DEGs between tumor and normal groups. The selection criteria for DEGs are *P* < 0.01, |log2FC| ≥ 2, and gene rank ≤ 10%. Then, we identified the overlapping DEGs between them using a Venn diagram (http://bioinformatics.psb.ugent.be/webtools/Venn/) [25].

### 2.2. Functional Analysis and Pathway Enrichment Analysis

Metascape (http://metascape.org/) is an online resource for gene annotation and analysis [26]. In the present study, Metascape was used to perform gene ontology (GO) and pathway analyses of 69 common hub genes. Pathway and process enrichment analyses were conducted based on several sources, including GO biological processes, The Kyoto Encyclopedia of Genes and Genomes pathways, reactome gene sets, and CORUM. Terms with a *P* value less than 0.01, a minimum count of 3, and an enrichment factor greater than 1.5 were considered to represent significant processes or pathways.

### 2.3. Construction of PPI Network and Identification of Hub Genes

We evaluated PPI information of common genes using the STRING online database (https://string-db.org/cgi/input.pl) and then visualized the resulting interaction network using Cytoscape software (http://www.cytoscape.org/) [27, 28]. A confidence score greater than 0.4 was defined as significant. The Molecular Complex Detection (MCODE) plugin in Cytoscape was used to further screen key genes in the PPI network with degree cutoff = 5, *K*‐score = 2, and node score cutoff = 0.2.

### 2.4. Development of Prognosis-Related Model

We downloaded gene expression profile and clinical data from TCGA (https://portal.gdc.cancer.gov/). To reduce statistical bias, BLCA patients were excluded if clinical information or overall survival (OS) was missing from their records. The prognostic value of 11 identified hub DEGs that were analyzed using the R survival package [29]. The DEGs with significant prognostic value were selected for further analysis. Based on these prognosis-related DEGs, least absolute shrinkage and selection operator (LASSO) Cox regression analysis was applied to establish prognostic model. Patients were then divided into high- and low-risk groups according to median risk score. Receiver operating characteristic (ROC) curve was used to assess the predictive accuracy of each gene and risk score. Univariate and multivariate Cox regression analysis was performed for independent analysis with other clinical characteristics. Nomogram was then used to assess 1-year, 3-year, and 5-year overall survival.

### 2.5. Validation of Prognostic Value of Three Key DEGs

PROGgenesV2 (http://genomics.jefferson.edu/proggene/filter.php) is a web resource that allows researchers to study the correlations between genes and overall survival (OS) in multiple cancers based on TCGA and GEO data [30]. PrognoScan (http://www.prognoscan.org/) was used to evaluate the associations between gene expression and patient prognosis, according to measures including OS and disease-free survival (DFS), across a large collection of publicly available cancer microarray datasets [31]. The OSblca database (http://bioinfo.henu.edu.cn/BLCA/BLCAList.jsp) provides a useful tool to assess novel prognostic biomarkers in bladder cancer, based on data from 1,075 bladder cancer patients, including OS, disease-specific survival (DSS), disease-free interval, and progression-free interval [32]. In this study, we further confirmed the prognostic value of key genes based on the abovementioned three databases. The hub genes identified in this way were defined as key prognosis-related genes.

### 2.6. Association between Prognosis-Related Key Genes and Clinical Characteristics

The UALCAN database (http://ualcan.path.uab.edu/) is a comprehensive web resource for analyzing cancer OMICS data (TCGA and MET500) [33, 34]. TISIDB (http://cis.hku.hk/TISIDB) is a publicly available resource that allows the user to explore the function of a gene and its role in tumor-immune features. TISIDB consists of 10 modules: function, literature, screening, immunotherapy, lymphocyte, immunomodulator, chemokine, subtype, clinical, and drug [35]. A previous study reported that the expression and prognostic values of DEGs were associated with clinical characteristics, including TNM stage, smoking history, lymph invasion, histological type, and immune subtype [5, 36]. Therefore, we explored the relationship between three key prognosis-related genes and clinical characteristics using UALCAN and TISIDB database.

### 2.7. Immune Cell Infiltration Analysis

TIMER is a user-friendly web portal for the systematic analysis of immune infiltrates across different types of cancer (https://cistrome. shinyapps.io/timer/) [37]. In this study, we used TIMER and TISIDB to analyze the associations between the three key genes and immune cell infiltration. *P* < 0.05 was considered as statistically significant.

### 2.8. Evaluation of the Value of Prognostic-Related Genes in Response to Immune Checkpoint Blockade

Previous studies have suggested that TMB and PD-L1 expressions are correlated with response to atezolizumab in mUC [20]. In this study, the correlation between prognostic-related gene expression and TMB score was calculated using Spearman's correlation [24, 38, 39]. The immunomodulator module of TISIDB was used to examine the associations between PDL1 and selected genes. Moreover, we used the screening module of TISIDB to explore whether the expression of prognosis-related genes showed significant differences between responders and nonresponders to immunotherapy. Tumor Immune Dysfunction and Exclusion (TIDE, http://tide.dfci.harvard.edu) algorithm was then performed to estimate custom biomarker predictive power of response outcome and overall survival [23, 40].

### 2.9. Exploration of the Model in the Tumor Immune Microenvironment and Immunotherapeutic Treatment

RNA sequence transcription data, mutation data, and relevant clinical information of BLCA patients were obtained from the TCGA (https://cancergenome.nih.gov/) database [41]. Immune function was analyzed based on three prognostic gene models using R package limma, GSVA, GSEABase, pheatmap, and reshape2 [42]. We used the TIDE algorithm to predict immunotherapy response [43].

### 2.10. Correlation between Prognostic-Related Genes and Their Target Drug

Gene Set Cancer Analysis (GSCALite, http://bioinfo.life.hust.edu.cn/web/GSCALite/) is a web server for the analysis a set of genes in cancers with different function modules [44]. In this study, we analyzed the drugs in the Drug Bank database targeting these three genes using the drug module of TISIDB. We further applied GSCALite to analyze the drug sensitivity of key ceRNA signatures.

## 3. Results

### 3.1. Identification of Hub Genes

**Figure 1:**
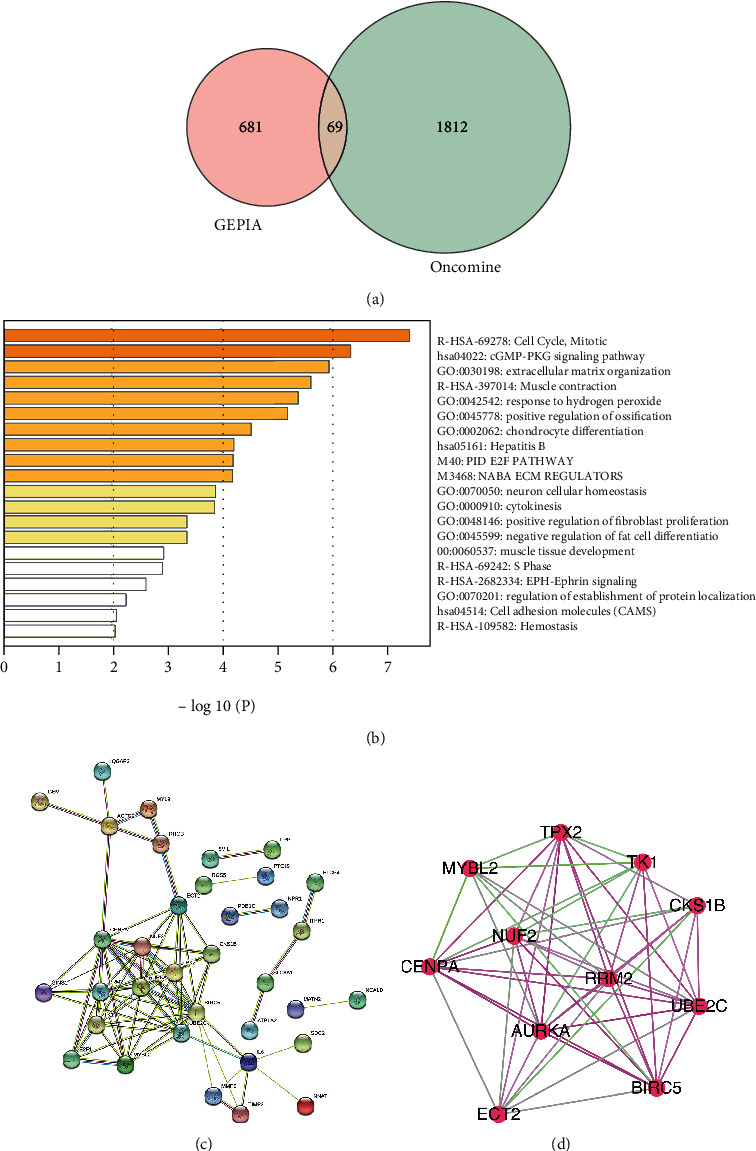
Identification of 11 hub genes. (a) Identification of common genes between GEPIA and Oncomine by Venn diagram. (b) Enriched terms of common genes identified by Metascape. Network of enriched terms colored by cluster ID. (c) PPI network of DEGs constructed with STRING software: nodes represent proteins; continuous lines represent direct interactions (physical), while indirect ones (functional) are represented by interrupted lines; and line thickness indicates the strength of data support. (d) Identification of hub genes using MCODE. Upregulated genes are represented by red nodes, while downregulated genes are denoted by green nodes. Node size is positively correlated with *P* value. The line color is determined by the combined score provided by STRING.

A total of 750 DEGs were identified from the GEPIA database, and 1,881 DEGs were identified from Oncomine. Sixty-nine common genes were screened out using the Venn diagram (Supplementary 1, Supplementary 2, Figure 1(a)). We then performed GO and pathway enrichment analyses for the common genes using Metascape. The results showed that these common genes were involved in 20 main GO terms and pathways, of which the top five were mitotic cell cycle, cGMP-PKG signaling pathway, extracellular matrix organization, muscle contraction, and response to hydrogen peroxide (Figure 1(b)). Finally, a PPI network of DEGs was constructed using the STRING and Cytoscape software, containing 69 nodes and 83 edges (Figure 1(c)). One significant module was identified using MCODE. This module contained 11 genes, Aurora-A kinase (AURKA), BIRC5, CENPA, CKS1B, ECT2, MYBL2, NUF2, RRM2, TK1, TPX2, and UBE2C, which were defined as hub genes (Figure 1(d)).

### 3.2. Construction of Prognostic Gene Model

**Figure 2:**
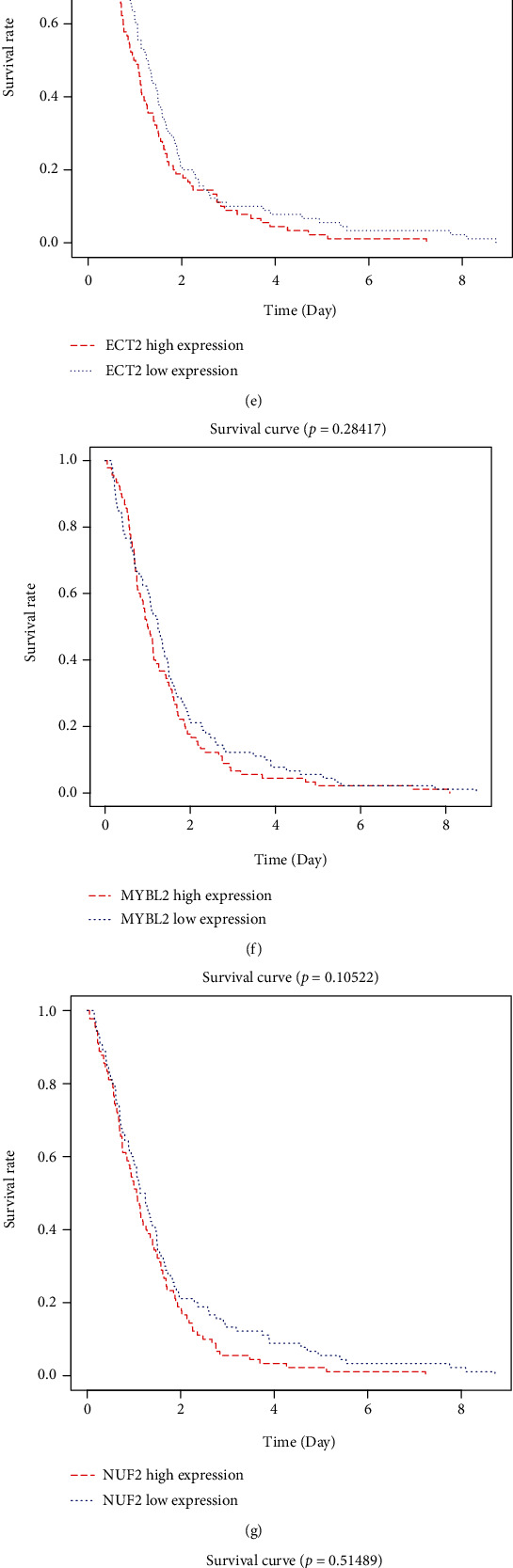
Overexpression of three genes is correlated with poor prognosis. (a)–(k) Associations between the expression of 11 hub genes and OS, evaluated using the R survival package. (a) AURKA, (b) BIRC5, (c) CENPA, (d) CKS1B, (e) ECT2, (f) MYBL2, (g) NUF2, (h) RRM2, (i) TK1, (j) TPX2, and (k) UBE2C. Only three key genes were associated with prognosis in BLCA.

**Figure 3:**
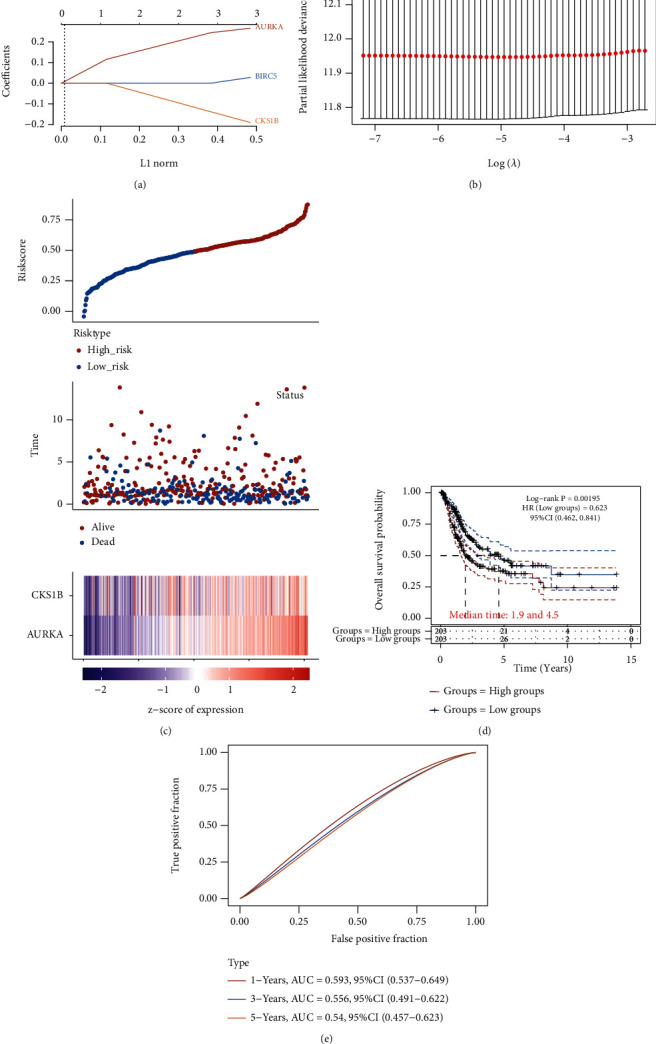
Construction of a prognostic model based on three key gene. (a) LASSO coefficient profiles of the three key genes. (b) Plots of the ten-fold cross-validation error rates. (c) Distribution of risk score, survival status, and the expression of three prognostic genes in BLCA. (d) Overall survival curves for BLCA patients in the high-/low-risk group. (e) ROC analysis was performed to measure the predictive value. BLCA: bladder urothelial carcinoma; LASSO: least absolute shrinkage and selection operator; ROC: receiver operating characteristic curve; HR: hazard ratio; CI: confidence interval.

The association between the expression of 11 hub genes and overall survival was evaluated using the R survival package. High expression of three genes (AURKA, BIRC5, and CKS1B) was related to an unfavorable prognosis (Figures 2(a)–2(k)). LASSO analysis was applied to establish a prognostic gene model based on these three prognostic DEGs (Figures 3(a) and 3(b)). The risk score = (0.214)∗AURKA + (−0.1054)∗CKS1B. The BLCA patients were divided into high- and low-risk score group based on risk score.  Figure 3(c) displayed the risk score distribution, survival status, and the expression of these genes. BLCA patients with high-risk score had an unfavorable prognosis than those with low-risk score (Figure 3(d)). ROC curve analysis indicated that the AUCs of the 1-, 3-, and 5-year prognosis models were 0.593, 0.556, and 0.54, respectively (Figure 3(e)).

### 3.3. Construction of Predictive Nomogram

**Figure 4:**
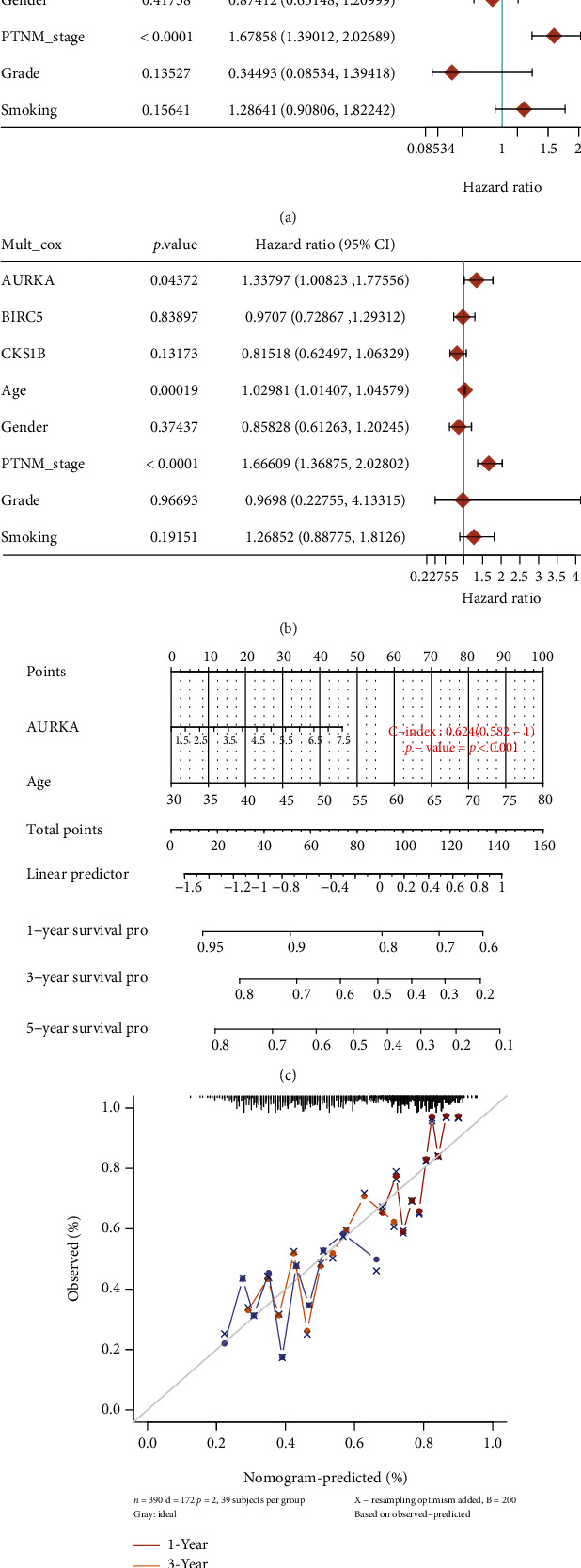
Nomogram for prediction of the outcome of BLCA patients. (a, b) Univariate and multivariate Cox regression analyses were applied to assess the independent predictive value of the three-gene signature. (c) Nomogram for prediction the 1-year, 3-year, and 5-year overall survival rate of BLCA patients. (d) The calibration plots of the nomogram. BLCA: bladder urothelial carcinoma.

According to the clinicopathologic features and three prognostic genes, we constructed a predictive nomogram to predict the survival probability (Figures 4(a) and 4(b)). The C-index of the nomogram was 0.624 (95% CI, 0.582-1). Calibration curves also showed a favorable predictive power of the nomogram (Figures 4(c) and 4(d)). Furthermore, we validated the prognostic value of the three genes in BLCA using the PROGgenesV2, PrognoScan, and OSblca databases. Our results showed that BLCA patients with higher expression levels of the three hub genes exhibited poorer OS, DFS, and DSS, indicating that the three hub genes may be associated with unfavorable prognosis (Table 1). In summary, our data suggested that the three key genes could serve as biomarkers of poor prognosis.

### 3.4. Association between Three Key Genes and Clinical Parameters in BLCA

**Figure 5:**
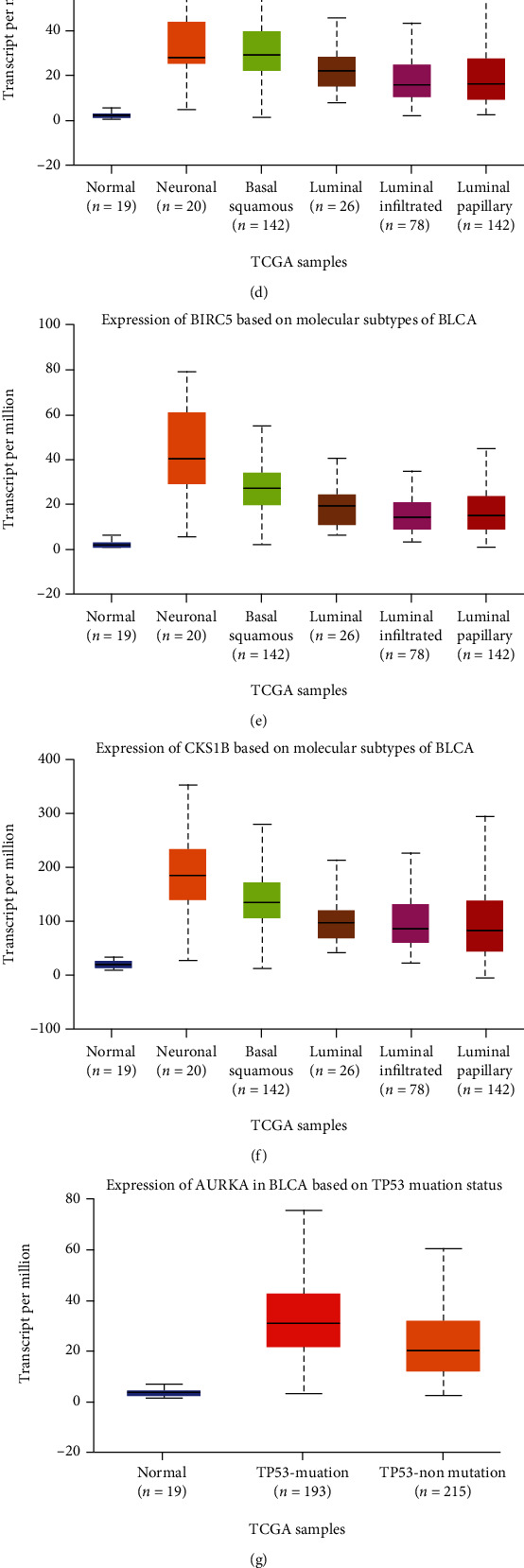
Evaluation of the association between three prognosis-related key genes and clinical factors. Expression of three key prognosis-related genes based on different sample types, according to (a)–(c) histological subtypes, (d)–(f) molecular subtypes, and (g)–(i) TP53 mutation status. (j)–(l) Associations between expression of three prognosis-related genes and immune subtypes across BLCA via TISIDB database: (j) AURKA, (k) BIRC5, and (l) CKS1B. Act_CD8: activated CD8 T cell; Tcm_CD8: central memory CD8 T cell; Tem_CD8: effector memory CD8 T; C1: wound healing; C2: IFN-*γ* dominant; C3: inflammatory; C4: lymphocyte depleted; C5: immunologically quiet; C6: TGF-*β* dominant. Kruskal–Wallis test was used to evaluate the statistical significance of differential expression.

A previous study reported that the OS of BLCA patients was significantly associated with clinical characteristics, including TNM stage, smoking history, lymph invasion, and histological type [32]. The three hub genes identified here were associated with various clinical characteristics including age, histological subtype, molecular subtype, nodal metastasis status, sample type, smoking, cancer stage, and TP53 mutation status (Table 2). Most importantly, overexpression of the three hub genes was positively correlated with histological subtypes (Figures 5(a)–5(c)). The expression of the three hub genes was higher in the basal squamous and neuronal subtypes than in the luminal subtype (Figures 5(d)–5(f)). BLCA patients with TP53 mutations also showed high expression of the three hub genes (Figures 5(g)–5(i)). Moreover, we found that the three prognosis-related genes had the highest expression levels in the C2 (IFN-gamma dominant) subtype and the lowest in the C3 (inflammatory) subtype (Figures 5(j)–5(l)). Taken together, these results suggest that increased expression of the three key genes might predict poor prognosis in patients with BLCA.

### 3.5. CD8+ T Cell Infiltration Predicts Poor Prognosis in BLCA

**Figure 6:**
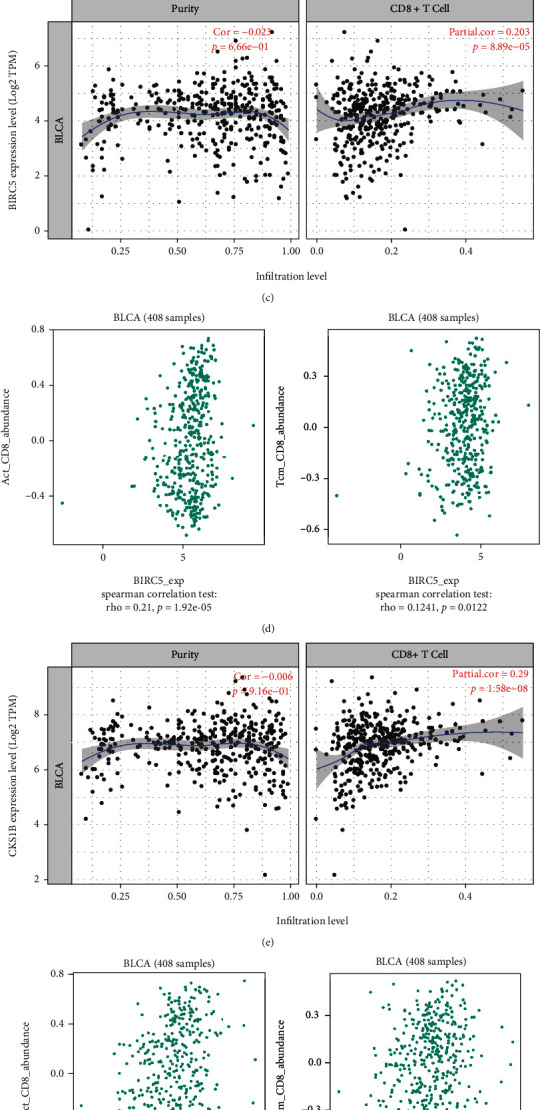
Association between three hub genes and immune infiltration. (a, b) Correlations of (a, b) AURKA, (c, d) BIRC5, and (e, f) CKS1B expression with immune infiltration level in BLCA. (g) Kaplan-Meier survival curves for different immune cells. Levels are divided into low and high by a defined slider. *P* value of log-rank test for comparing survival curves of the two groups is shown in each plot.

The TIMER and TISIDB databases were used to explore the relationship between the three prognosis-related genes and tumor-infiltrating immune cells. The three genes were positively associated with levels of infiltrating CD8+ T cells, neutrophils, and dendritic cells. Expression of BIRC5 was negatively correlated with infiltration of B cells (Table 3, Figures 6(a)–6(f)). High levels of infiltration of CD8+ T cells were associated with poor prognosis (Figure 6(g)). These results suggest that these genes may affect prognosis via regulation of CD8+ T cells.

### 3.6. Three Key Genes Correlated with ICB Response

**Figure 7:**
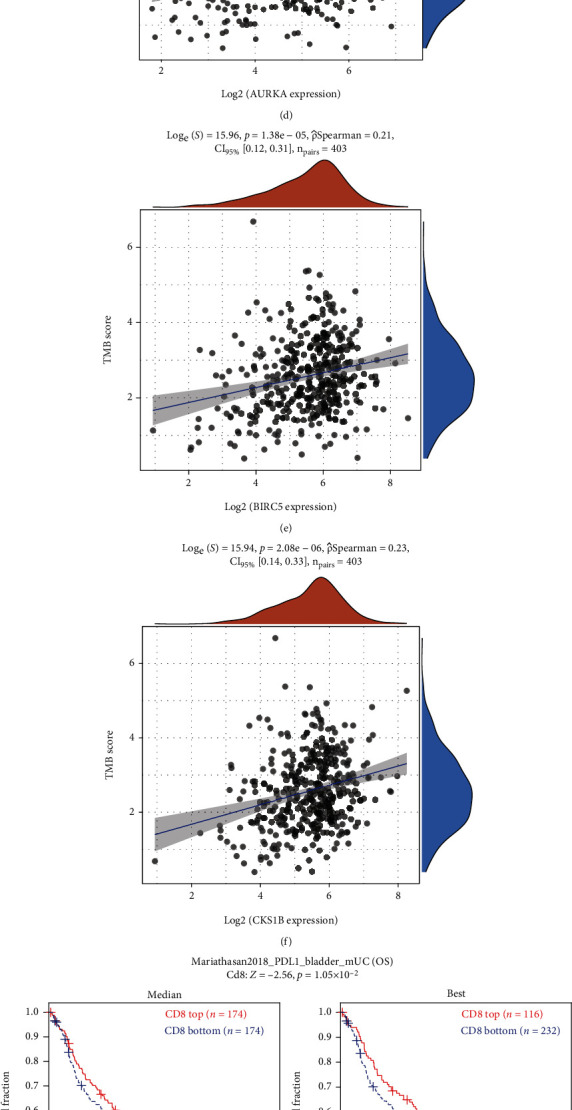
Evaluation of the value of three key genes in response to ICB. (a)–(c) Correlations between three hub genes and CD274 (PD-L1) mRNA expression. (a) AURKA, (b) BIRC5, and (c) CKS1B. (d)–(f) TMB shows positive relationships with three hub genes in BLCA. (d) AURKA, (e) BIRC5, and (f) CKS1B. (g)–(i) The association of the different (CD8 + T cell, CD274, and mutation) biomarker with patients' overall survival through Kaplan-Meier curves. (g) CD8 + T cell, (h) CD274, and (i) mutation.

**Figure 8:**
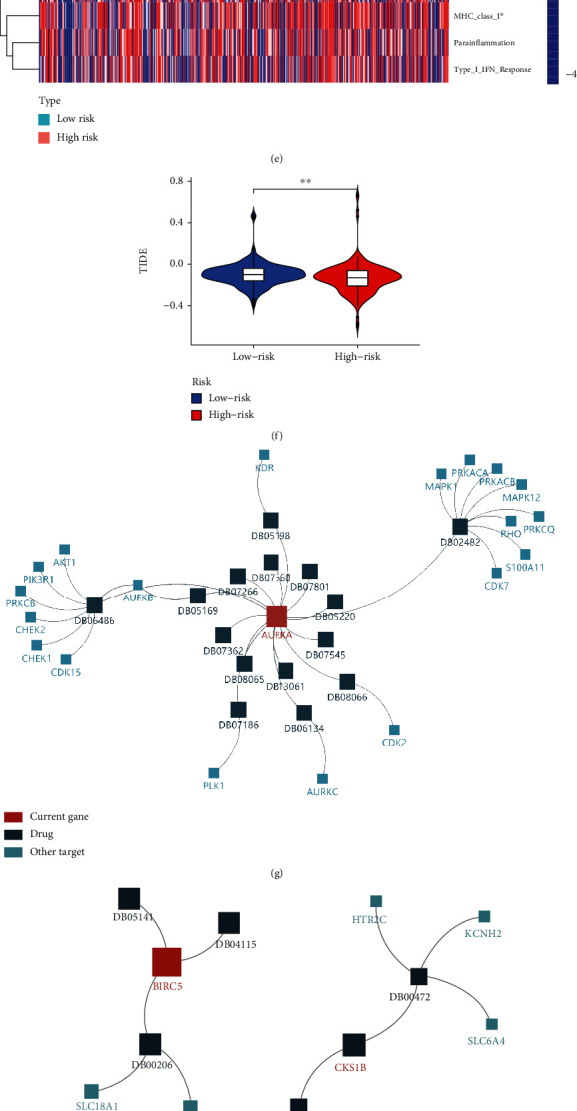
Estimation of the TME and cancer immunotherapy response and evaluation target drugs of these genes. Association between three genes and immunosuppressive indices (columns), including T cell dysfunction score and ICB survival outcome. (b)–(d) Survival differences between groups with high and low expression of biomarkers after anti-PDL1. (b) AURKA, (c) BIRC5, and (d) CKS1B. (e) Immune function difference between high-risk groups and low-risk group. (f) The difference in immunotherapy response between high- and low-risk groups based on the TIDE score. (g)–(i) Drugs targeting three key genes were collected from the DrugBank database: (g) AURKA, (h) BIRC5, and (i) CKS1B. Red rectangle represents the current gene, blue rectangle represents a drug, and green rectangle indicates other targets. (j, k) Correlation of three prognostic genes expression and drug sensitivity from GSCA database. (j) CTRP drug sensitivity and expression correlation. (k) GDSC drug sensitivity and expression correlation. GSCA: Gene Set Cancer Analysis; CTRP: Cancer Therapeutics Response Portal; GDSC: Genomics of Drug Sensitivity in Cancer; ICB: immune checkpoint blockade; OS: overall survival; TME: tumor immune microenvironment.

Our results indicated that the expression levels of the three hub genes were positively correlated with infiltration of PD-L1 (CD274) expression and TMB (Figures 7(a)–7(f)). We also found that high infiltration of CD8 + T cell, high expression of CD274, and high TMB were associated with prolonged overall survival after anti-PD-L1 therapy in BLCA (Figures 7(g)–7(i)). The association score of ICB survival outcome showed that three genes were correlated with ICB benefit in different cancer, especially in BLCA (Figures 8(a)–8(d)). Finally, our results showed that the three hub genes exhibited a significant difference in expression between responders and nonresponders to atezolizumab in urothelial cancer via TISIDB analysis (Table 4). Taken together, these results suggest that the impact of the three hub genes on response to immunotherapy in BLCA may be associated with TMB and PD-L1 expression. Our identified three key genes might serve as an indicator to evaluate response to ICB immunotherapy.

### 3.7. Evaluation of the Tumor Immune Microenvironment and Immunotherapy Response Based on Prognostic-Related Model

To explore the underlying molecular mechanisms of three gene-related model, we performed immune function enrichment analysis. The low-risk and high-risk groups displayed significant differences in the expression of immune indicators, including type II IFN response and MHC class I (Figure 8(e)). We further evaluated whether the risk model based on three key genes could be used to predict immunotherapy response in BLCA. Our result showed that the TIDE score of the high-risk group is less than that of the low-risk group, indicating that compared with the low-risk group, the high-risk group can benefit more from immunotherapy (Figure 8(f)).

### 3.8. Drug–Gene Interaction Network

Drugs targeting three key genes were collected from the Drug Bank database. AURKA and 18 other targets were correlated with 15 drugs. BIRC5 and two targets were related to 3 drugs. CKS1B and another 3 targets interacted with 2 drugs (Figures 8(g)–8(i)). Based on GSCALite analysis, we found that most drugs were effective in association with increased expression of AURKA both in CTRP and GDSC database, while BIRC5 was positively regulated by 2 drugs and negatively regulated by 2 drugs in GDSC database. Specifically, these molecules could be exploited as potential therapeutic drug targets for BLCA (Figures 8(j) and 8(k)).

## 4. Discussion

BLCA is one of the most common urinary cancers in the world, with high recurrence and mortality rates that limit the efficacy of treatment [45]. It is therefore essential to understand the molecular mechanism of BLCA. With the development of bioinformatics technology, increasing numbers of studies are using bioinformatics analysis to develop biomarkers and explore the molecular mechanism of BLCA [46, 47]. However, there are still no reliable biomarkers associated with prognosis of BLCA patients. In recent years, immunotherapy has attracted considerable attention owing to its influence on the treatment of locally advanced and metastatic BLCA, but the objective response rate of BLCA to immune checkpoint inhibitors (ICIs) was low [12, 48]. Therefore, it is critical to identify satisfactory signatures to effectively predict prognosis and evaluate the benefit of immunotherapy in BLCA patients.

AURKA is a member of the serine/threonine kinase family and has a role in regulation of the cell cycle [49]. Accumulating evidence indicates that AURKA is overexpressed in various cancers, including breast cancer, head and neck cancer, esophagus cancer, hematological malignancies, colorectal cancer, stomach cancer, pancreatic cancer, and ovarian and prostate cancers [50, 51]. Pathological overexpression of AURKA is correlated with shorter survival of cancer patients. According to previous reports, high expression of Aurora-A in tumor cells is closely related to poor prognosis [46, 51]. BIRC5 is a member of the inhibitor of apoptosis gene family, which has dual roles in promoting cell proliferation and preventing apoptosis [50]. Overexpression of BIRC5 has been reported in several malignancies, and higher BIRC5 expression was also found to be associated with decreased survival [52]. CKS1B is an oncogene that has been reported to show increased expression in various tumors [53]. In accordance with previous studies, our results demonstrated that high expression of three hub genes was associated with poor prognosis (Figures 2(a), 2(b), and 2(d)). Further analysis revealed that these three key genes were overexpressed in nonpapillary tumors, the basal squamous subtype, TP53 mutation patients, and C2 (IFN-gamma dominant) subtype (Figures 5(a)–5(l)). Three hub genes were highly correlated with TMB and PD-L1 expressions (Figures 7(a)–7(f)). Previous studies have reported that TP53 mutation is associated with poor prognosis [54]. TMB has prognostic roles in various cancer types, including BLCA [55]. High expression of PD-L1 is associated with worse cancer outcomes [19]. A previous study reported that the C2 subtype had the highest levels of CD8 + T cells, as well as having less favorable outcomes [36]. These results indicated that the expression of these three key genes may represent a marker of poor prognosis in BLCA, as well as in nonpapillary tumors, the basal squamous subtype, TP53 mutation patients, tumor with high TMB, and the C2 subtype.

Tumor immunity is an extremely complex biological process, and factors affecting the efficacy of immune checkpoint inhibitors (IMCIs) are associated with PD-L1 expression level, tumor-infiltrating lymphocytes (TILs), and tumor mutational burden (TMB) [13]. TMB has been investigated in various malignancies and found to be correlated with response to atezolizumab in mUC [20–22]. Increased CD8 + T cell infiltration has been reported to be correlated with better immunotherapeutic effect [56]. A previous study showed that biomarkers related to CD8 + T cell infiltration could facilitate the monitoring of immunotherapy response and the exploration of the immune infiltration mechanism in clear cell renal cell carcinoma. A previous study showed that biomarkers related to CD8 + T cell infiltration could facilitate the monitoring of immunotherapy response and the exploration of the immune infiltration mechanism in clear cell renal cell carcinoma. AURKA is overexpressed in cancer cells but not in normal tissues, making it a potential target for immunotherapy. AURKA-specific CD8(+) T cells can selectively lyse leukemia cells [21]. CD8 + cytotoxic T lymphocytes (CTLs) generated in vitro can recognize AURKA epitope (YLILEYAPL). In addition, these CTLs can kill leukemia cells endogenously expressing AURKA, indicating that the homologous epitopes are naturally processed and presented at a level sufficient for immunotherapeutic applications [57]. BIRC5 is a member of the apoptosis inhibitor gene family, which regulates several cancers by activating cell apoptosis process [58]. BIRC5 is correlated with T cell survival and proliferation, which can increase the accumulation and persistence of CD8(+) T cells following an encounter with Ag [59]. CKS1B is associated with cell cycle. The association between CKS1B and ICI is rare.

To further explore the molecular mechanism of three genes related model. We found that immune function was different between high- and low-risk groups (Figure 8(e)). Our result indicated that three hub genes were highly correlated with CD8 + T cell, TMB, and PD-L1 expression (Figures 6(a)–6(f) and 7(a)–7(f)). We further found that CD8 + T cell, TMB, and PD-L1 expression were positively correlated with OS after anti-PDL1 therapy (Figures 7(g)–7(i)). The association score of ICB survival outcome showed that three genes were correlated with ICB benefit (Figures 8(a)–8(d)). In addition, we found that three hub genes exhibited a significant difference in expression between responders and nonresponders to atezolizumab in urothelial cancer via TISIDB analysis (Table 4). Studies showed that TIDE score can effectively predict the response of immunotherapy [23, 42]. In this study, TIDE score analysis suggested that patients with high-risk score get a better response to immunotherapy. Taken together, we can infer that the expression of three key genes and their related model may provide reliable biomarkers to evaluate the response to immunotherapy, and the mechanism of three genes in ICB response remains to be further studied. Finally, we developed potential drug targets that interact with three genes (Figures 8(g)–8(k)). Collectively, these data suggest that three prognostic genes were correlated with ICB response in BLCA, which may be associated with CD8 + T cells, TMB, and PD-L1 expression. Drug–gene interaction network analysis indicated that these genes and their related drugs could be used in the development of new targets for BLCA immunotherapy.

## 5. Conclusions

In summary, three key genes in BLCA were found to be correlated with poor prognosis and immunotherapy response in BLCA. Three prognostic genes and their related drugs may help to develop new targets for improving BLCA immunotherapy. Combination of three genes' inhibitor and anti-PDL1 may provide new insights for improving effectiveness of immunotherapy. Nevertheless, the present study has certain limitations. For example, our experimental design mainly focuses on the computational nature. In fact, no validation analysis has been performed based on BLCA cell lines or clinical samples, which greatly limit the impact of our results. Moreover, the biological mechanism of three gene-related model has not been fully elucidated. We will perform external experiments based on BLCA cell lines or clinical samples to support our results and investigate the underlying molecular mechanism of three genes in prediction immunotherapy response in our following work.

## Figures and Tables

**Table 1:** Confirmation of the associations of three hub genes with prognosis in three different databases. PROGgeneV2, PrognoScan, and OSblca databases were used to confirm the prognostic value of three hub genes in BLCA. HR: hazard ratio.

Database	Dataset	Gene	Endpoint	*P* value	HR [95% CI low-CI up]
PROGgenesV2	GSE13507	AURKA	OS	0.00135	1.39 [1.14-1.7]
	GSE13507	BIRC5	OS	0.01299	1.94 [1.15-3.26]
	GSE13507	CKS1B	OS	0.03256	1.24 [1.02-1.5]
	GSE19915	BIRC5	OS	0.00009	2.62 [1.62-4.24]

PrognoScan	GSE13507_ILMN_1680955	AURKA	OS	0.00128	1.39 [1.14-1.70]
	GSE13507_ILMN_1680955	AURKA	DFS	0.00011	1.91 [1.40-2.62]
	GSE5287_210334_x_at	BIRC5	OS	0.00560	7.68 [2.55-23.14]
	GSE5287_202095_s_at	BIRC5	OS	0.00183	2.43 [1.35-4.38]
	GSE13507_ILMN_1710082	BIRC5	OS	0.00179	1.94 [1.15-3.26]
	GSE13507_ILMN_1710082	BIRC5	DFS	0.00077	3.22 [1.65-6.31]
	GSE13507_ILMN_1719256	CKS1B	DFS	0.04721	1.56 [1.17-2.08]

OSblca	GSE13507_ILMN_1710082	BIRC5	OS	0.01880	1.837 [1.1059-3.0515]
	GSE13507_ILMN_1680955	AURKA	OS	0.00400	2.1412 [1.2742-3.5983]
	GSE19915	BIRC5	DSS	0.00030	4.4378 [1.9813-9.9398]
	GSE32548_ILMN_2349459	BIRC5	OS	0.04900	2.2344 [1.0036-4.9749]
	GSE48507_ILMN_2349459	BIRC5	OS	0.00400	2.5862 [1.3551-4.936]
	GSE48075_ILMN_1803124	BIRC5	OS	0.00900	2.3966 [1.2444-4.6154]
	GSE32548_ILMN_1719256	CKS1B	OS	0.03500	2.3667 [1.026-5.2711]
	GSE32548_ILMN_2041046	CKS1B	OS	0.00790	2.9205 [1.3245-6.4394]

**Table 2:** Relationships of three prognostic genes with clinical characteristics. Clinical characteristics included age, histological subtype (papillary or nonpapillary tumor), molecular subtype (luminal papillary, luminal infiltrated, luminal, basal squamous, and neuronal), nodal metastasis status (N0: no regional lymph node metastasis; N1: metastases in 1–3 axillary lymph nodes; N2: metastases in 4–9 axillary lymph nodes; N3: metastases in 10 or more axillary lymph nodes), sample type, smoking, cancer stage, and TP53 mutation status.

Gene symbol	Clinical characteristic	Comparison	*P* value
AURKA	Age	Normal-vs-age (41-60 Y)	4.00E-15
		Normal-vs-age (61-80 Y)	<1E-12
		Normal-vs-age (81-100Y)	7.10E-10

BIRC5	Age	Normal-vs-age (41-60 Y)	1.82E-10
		Normal-vs-age (61-80 Y)	3.99991E-12
		Normal-vs-age (81-100Y)	2.40E-09

CKS1B	Age	Normal-vs-age (41-60 Y)	8.88E-16
		Normal-vs-age (61-80 Y)	1.62E-12
		Normal-vs-age (81-100Y)	2.617E-07

AURKA	Histological subtypes	Normal-vs-papillary tumors	6.55E-15
		Normal-vs-nonpapillary tumors	<1E-12
		Papillary tumors-vs-nonpapillary tumors	3.00E-03

BIRC5	Histological subtypes	Normal-vs-papillary tumors	6.40E-07
		Normal-vs-nonpapillary tumors	8.40E-10
		Papillary tumors-vs-nonpapillary tumors	1.10E-03

CKS1B	Histological subtypes	Normal-vs-papillary tumors	1.77E-04
		Normal-vs-nonpapillary tumors	3.27E-08
		Papillary tumors-vs-nonpapillary tumors	2.38E-04

AURKA	Molecular subtypes	Normal-vs-neuronal	1.54E-06
		Normal-vs-basal squamous	<1E-12
		Normal-vs-luminal	2.26E-10
		Normal-vs-luminal_infiltrated	1.49E-11
		Normal-vs-luminal_papillary	1.67E-12
		Neuronal-vs-luminal	1.05E-02
		Neuronal-vs-luminal_infiltrated	2.48E-03
		Neuronal-vs-luminal_papillary	6.00E-03
		Basal squamous-vs-luminal	1.66E-05
		Basal squamous-vs-luminal_infiltrated	8.65E-12
		Basal squamous-vs-luminal_papillary	1.11E-09

BIRC5	Molecular subtypes	Normal-vs-neuronal	3.20E-07
		Normal-vs-basal squamous	1.65E-12
		Normal-vs-luminal	2.93E-07
		Normal-vs-luminal_infiltrated	4.66E-07
		Normal-vs-luminal_papillary	1.15E-08
		Neuronal-vs-basal squamous	3.40E-03
		Neuronal-vs-luminal	9.81E-05
		Neuronal-vs-luminal_infiltrated	4.18E-05
		Neuronal-vs-luminal_papillary	9.99E-05
		Basal squamous-vs-luminal	3.10E-06
		Basal squamous-vs-luminal_infiltrated	1.03E-11
		Basal squamous-vs-luminal_papillary	1.36E-08

CKS1B	Molecular subtypes	Normal-vs-neuronal	9.74E-07
		Normal-vs-basal squamous	1.62E-12
		Normal-vs-luminal	2.40E-08
		Normal-vs-luminal_infiltrated	5.97E-11
		Normal-vs-luminal_papillary	4.44E-15
		Neuronal-vs-basal squamous	4.57E-02
		Neuronal-vs-luminal	1.74E-03
		Neuronal-vs-luminal_infiltrated	1.46E-03
		Neuronal-vs-luminal_papillary	9.79E-04
		Basal squamous-vs-luminal	9.60E-04
		Basal squamous-vs-luminal_infiltrated	9.92E-05
		Basal squamous-vs-luminal_papillary	2.26E-07

AURKA	Nodal metastasis status	Normal-vs-N0	1.62E-12
		Normal-vs-N1	1.69E-12
		Normal-vs-N2	2.22E-12
		Normal-vs-N3	1.25E-10

BIRC5	Nodal metastasis status	Normal-vs-N0	1.34E-11
		Normal-vs-N1	1.69E-10
		Normal-vs-N2	8.57E-09
		Normal-vs-N3	7.04E-03
		N1-vs-N2	2.50E-02

CKS1B	Nodal metastasis status	Normal-vs-N0	1.62E-12
		Normal-vs-N1	1.04E-09
		Normal-vs-N2	1.63E-12
		Normal-vs-N3	8.60E-04
		N0-vs-N1	4.35E-02
		N1-vs-N2	1.70E-02
		N2-vs-N3	2.42E-02

AURKA	Sample types	Normal-vs-primary	1.62E-12
BIRC5		Normal-vs-primary	5.35E-11
CKS1B		Normal-vs-primary	1.62E-12

AURKA	Smoking habit	Normal-vs-nonsmoker	1.24E-14
		Normal-vs-smoker	1.63E-12
		Normal-vs-reformed smoker1	<1E-12
		Normal-vs-reformed smoker2	1.33E-15
		Nonsmoker-vs-reformed smoker1	8.60E-03
		Nonsmoker-vs-reformed smoker2	9.46E-03
BIRC5	Smoking habit	Normal-vs-nonsmoker	1.82E-09
		Normal-vs-smoker	2.35E-10
		Normal-vs-reformed smoker1	3.83E-12
		Normal-vs-reformed smoker2	3.34E-12

CKS1B	Smoking habit	Normal-vs-nonsmoker	1.63E-12
		Normal-vs-smoker	5.55E-16
		Normal-vs-reformed smoker1	<1E-12
		Normal-vs-reformed smoker2	1.62E-12

AURKA	Cancer stage	Normal-vs-stage2	1.62E-12
		Normal-vs-stage3	1.62E-12
		Normal-vs-stage4	1.62E-12

BIRC5	Cancer stage	Normal-vs-stage2	1.53E-10
		Normal-vs-stage3	3.81E-13
		Normal-vs-stage4	7.86E-12

CKS1B	Cancer stage	Normal-vs-stage2	<1E-12
		Normal-vs-stage3	<1E-12
		Normal-vs-stage4	1.62E-12

AURKA	TP53 mutation status	Normal-vs-TP53-mutant	1.62E-12
		Normal-vs-TP53-nonmutant	3.63E-13
		TP53-mutant-vs-TP53-nonmutant	3.80E-12

BIRC5	TP53 mutation status	Normal-vs-TP53-mutant	1.63E-12
		Normal-vs-TP53-nonmutant	8.02E-09
		TP53-mutant-vs-TP53-nonmutant	7.83E-08

CKS1B	TP53 mutation status	Normal-vs-TP53-mutant	1.62E-12
		Normal-vs-TP53-nonmutant	8.44E-15
		TP53-mutant-vs-TP53-nonmutant	2.20E-12

**Table 3:** Associations of three prognosis-related genes with immune cell infiltration by TIMER.

Gene	Immune cell	Cor	*P* value
AURKA	CD8 + T cell	0.288875857	1.82E-08
	Neutrophil	0.166799639	0.001425828
	Dendritic cell	0.317303773	5.56E-10

BIRC5	B cell	-0.105547337	0.044470614
	CD8 + T cell	0.203404473	8.89E-05
	Neutrophil	0.108824791	0.038228327
	Dendritic cell	0.299191494	5.53E-09

CKS1B	CD8 + T cell	0.290098344	1.58E-08
	Neutrophil	0.12429351	0.017829713
	Dendritic cell	0.302158923	3.83E-09

**Table 4:** Differences in expression of three genes between responders and nonresponders.

Gene symbol	Description	Drug	AveExpr	*P*
AURKA	Aurora kinase A	Anti-PD-L1 (atezolizumab)	5.632	0.0007978
BIRC5	Baculoviral IAP repeat containing 5	Anti-PD-L1 (atezolizumab)	2.755	0.0001359
CKS1B	CDC28 protein kinase regulatory subunit 1B	Anti-PD-L1 (atezolizumab)	1.739	0.001768

## Data Availability

The data that support the findings of this study are included in the article/Supplementary Material, and further inquiries can be directed to the corresponding author.

## Abbreviations

BLCA: Bladder urothelial carcinoma
DFS: Disease-free survival
DSS: Disease-specific survival
GEPIA: Gene expression profiling interactive analysis
GO: Gene ontology
IMCIs: Immune checkpoint inhibitors
mUC: Metastatic urothelial cancer
OS: Overall survival
PPI: Protein–protein interaction
TIIC: Tumor-infiltrating immune cell
TMB: Tumor mutation burden
TIDE: Tumor immune dysfunction and exclusion
TME: Tumor immune microenvironment.

## Supplementary Materials

Supplementary Materials Supplementary 1: different expression genes from GEPIA database. Supplementary 2: different expression genes from Oncomine database. Supplementary 3: original source data: the relevant code or R software.
