# Supplementary material for: Three Prognostic Biomarkers Correlate with Immune Checkpoint Blockade Response in Bladder Urothelial Carcinoma
Source: Int J Genomics. 2022 May 26;2022:3342666. doi: 10.1155/2022/3342666 (PMC9162857; doi:10.1155/2022/3342666)
Supplement: Supplementary Materials — Supplementary 1: different expression genes from GEPIA database. Supplementary 2: different expression genes from Oncomine database. Supplementary 3: original source data: the relevant code or R software. [file 3342666.f1.zip › Supplemetary 1-GEPIA-Diff (1).pdf]

Supplementary 1 Different expression genes from GEPIA database

Gene Symbol	Gene ID	Median (Tumor)	Median (Normal)	Log2(Fold Change)	adjp
AC239868.2	ENSG00000280874.1	35.69	0	5.197	9.74E-13
AC239868.3	ENSG00000281134.1	35.626	0	5.195	2.76E-12
UBE2C	ENSG00000175063.16	130.734	3.811	4.775	1.40E-39
RP5-940J5.9	ENSG00000269968.1	82.733	2.166	4.725	3.95E-03
CDC20	ENSG00000117399.13	68.554	2.854	4.174	6.93E-33
MYBL2	ENSG00000101057.15	36.015	1.179	4.086	1.52E-23
TOP2A	ENSG00000131747.14	52.916	2.752	3.845	7.01E-32
FAM83A	ENSG00000147689.16	21.87	0.64	3.802	8.03E-03
TROAP	ENSG00000135451.12	28.819	1.18	3.774	5.34E-30
KRT7	ENSG00000135480.14	1655.555	121.718	3.755	1.79E-07
CTB-191K22.5	ENSG00000267815.1	53.79	3.119	3.734	2.88E-07
TK1	ENSG00000167900.11	69.738	4.572	3.666	7.46E-31
BIRC5	ENSG00000089685.14	29.485	1.424	3.653	1.19E-26
AURKB	ENSG00000178999.12	34.675	1.95	3.596	2.06E-27
CENPF	ENSG00000117724.12	32.095	1.748	3.59	7.40E-21
ARAP1-AS1	ENSG00000256007.1	18.904	0.687	3.561	1.98E-09
TPX2	ENSG00000088325.15	33.049	1.885	3.561	5.35E-24
FOXM1	ENSG00000111206.12	29.565	1.6	3.555	2.38E-20
RRM2	ENSG00000171848.13	37.425	2.306	3.539	4.46E-17
PKMYT1	ENSG00000127564.16	27.84	1.58	3.483	5.31E-31
CCNB2	ENSG00000157456.7	30.939	1.907	3.458	2.12E-25
KRT16	ENSG00000186832.8	32.895	2.235	3.389	9.21E-03
RP11-316M1.12	ENSG00000259357.2	23.095	1.397	3.329	3.68E-10
CA9	ENSG00000107159.12	10.455	0.235	3.213	1.93E-06
CDK1	ENSG00000170312.15	35.559	2.985	3.198	2.31E-31
CCNB1	ENSG00000134057.14	59.941	5.693	3.187	1.05E-22
KIF20A	ENSG00000112984.11	14.325	0.685	3.185	1.79E-21
RP11-498C9.3	ENSG00000262413.1	8.795	0.08	3.182	4.97E-04
NCAPH	ENSG00000121152.9	14.775	0.757	3.166	3.14E-22
MELK	ENSG00000165304.7	13.23	0.612	3.142	1.24E-17
ZWINT	ENSG00000122952.16	36.459	3.293	3.125	1.20E-22
KIF2C	ENSG00000142945.12	18.385	1.242	3.112	3.76E-24
NUSAP1	ENSG00000137804.12	26.905	2.255	3.1	3.17E-19
GJB2	ENSG00000165474.5	37.829	3.556	3.091	8.20E-05
NUF2	ENSG00000143228.12	12.595	0.61	3.078	4.47E-19
TUBB3	ENSG00000258947.6	35.407	3.314	3.077	1.21E-08
AC098973.2	ENSG00000225548.5	7.429	0	3.075	8.14E-04
NEK2	ENSG00000117650.12	12.115	0.573	3.06	4.24E-20
AC021066.1	ENSG00000281415.1	294.709	34.486	3.059	3.29E-03
RP4-738P15.6	ENSG00000279322.1	20.559	1.627	3.037	2.08E-09

AC104534.3	ENSG00000268083.5	8.89	0.221	3.018	2.41E-04
CENPA	ENSG00000115163.14	11.4	0.54	3.009	8.13E-20
CDCA3	ENSG00000111665.11	28.406	2.658	3.007	3.43E-24
CDCA5	ENSG00000146670.9	17.71	1.345	2.996	2.09E-19
CST1	ENSG00000170373.8	8.2	0.154	2.995	2.84E-07
CDCA8	ENSG00000134690.10	14.965	1.023	2.98	9.83E-22
PTTG1	ENSG00000164611.12	69.915	8.129	2.958	2.01E-20
KIAA0101	ENSG00000166803.10	51.46	5.785	2.951	3.77E-26
ASF1B	ENSG00000105011.8	20.659	1.819	2.942	1.21E-25
UHRF1	ENSG00000276043.4	14.465	1.036	2.925	1.96E-24
PLK1	ENSG00000166851.14	26.015	2.586	2.913	5.66E-18
MKI67	ENSG00000148773.12	17	1.402	2.906	2.06E-20
CEP55	ENSG00000138180.15	13.125	0.923	2.877	7.46E-19
CDC45	ENSG00000093009.9	14.655	1.143	2.869	1.96E-26
MMP1	ENSG00000196611.4	11.055	0.662	2.858	3.43E-05
TMEM132A	ENSG00000006118.14	37.236	4.28	2.856	2.54E-23
AURKA	ENSG00000087586.17	18.775	1.755	2.844	1.03E-19
GGH	ENSG00000137563.11	40.975	5	2.807	5.02E-13
ANLN	ENSG00000011426.10	16.72	1.54	2.802	6.77E-13
CDT1	ENSG00000167513.8	13.995	1.17	2.789	1.33E-21
RECQL4	ENSG00000160957.12	27.77	3.165	2.788	3.70E-28
CTC-425F1.4	ENSG00000267458.1	34.5	4.149	2.785	4.73E-06
DUXAP8	ENSG00000206195.10	8.99	0.453	2.781	7.34E-10
KIFC1	ENSG00000237649.7	21.495	2.284	2.776	3.94E-21
LINC01296	ENSG00000244306.9	8.405	0.39	2.758	3.21E-09
RP11-256P1.1	ENSG00000249971.1	5.765	0	2.758	6.88E-05
PRC1	ENSG00000198901.13	44.835	5.835	2.746	4.89E-19
DLGAP5	ENSG00000126787.12	8.595	0.448	2.728	5.95E-16
SPP1	ENSG00000118785.13	50.83	6.861	2.721	2.08E-06
KLHDC7B	ENSG00000130487.5	7.505	0.29	2.721	1.03E-04
KIF18B	ENSG00000186185.13	7.205	0.26	2.703	3.50E-19
SPAG5	ENSG00000076382.16	31.465	3.988	2.702	1.67E-24
FAM64A	ENSG00000129195.15	8.615	0.487	2.693	3.69E-15
RP11-667K14.4	ENSG00000262533.1	8.45	0.465	2.69	3.33E-07
PYCR1	ENSG00000183010.16	36.63	4.854	2.685	1.40E-11
ISG15	ENSG00000187608.8	211.842	32.513	2.667	9.72E-11
RP11-161H23.5	ENSG00000258232.2	283.145	43.736	2.667	1.94E-27
IQGAP3	ENSG00000183856.10	7.475	0.335	2.666	8.99E-18
APOC1	ENSG00000130208.9	102.963	15.507	2.655	3.60E-12
TRIP13	ENSG00000071539.13	12.405	1.155	2.637	4.00E-14
FUT3	ENSG00000171124.12	13.855	1.391	2.635	3.58E-04
CENPM	ENSG00000100162.14	20.659	2.499	2.63	1.26E-25
NDC80	ENSG00000080986.12	12.19	1.153	2.615	6.04E-15

KIF23	ENSG00000137807.13	13.735	1.405	2.615	3.62E-16
LY6K	ENSG00000160886.13	21.069	2.604	2.614	9.44E-06
UBE2T	ENSG00000077152.9	31.659	4.342	2.612	1.16E-22
HN1	ENSG00000189159.15	418.112	67.817	2.607	2.23E-32
RAD54L	ENSG00000085999.11	9.61	0.75	2.6	1.55E-22
NCAPG	ENSG00000109805.9	8.7	0.6	2.6	8.02E-18
CKS2	ENSG00000123975.4	101.359	15.952	2.594	7.99E-23
CST6	ENSG00000175315.2	14.775	1.615	2.593	8.75E-08
CCNA2	ENSG00000145386.9	14.205	1.54	2.582	5.53E-15
KRT14	ENSG00000186847.5	12.009	1.177	2.579	3.37E-03
KRT17	ENSG00000128422.15	1036.137	173.087	2.575	7.41E-04
AP000349.2	ENSG00000280178.1	8.625	0.62	2.571	2.52E-08
KIF4A	ENSG00000090889.11	7.49	0.428	2.571	2.83E-17
LL22NC03-N14H11.1	ENSG00000272872.1	7.26	0.395	2.566	1.01E-09
CDC6	ENSG00000094804.9	10.72	1.004	2.548	2.16E-15
SPC25	ENSG00000152253.8	8.085	0.553	2.548	1.64E-15
FABP6	ENSG00000170231.15	12.435	1.34	2.522	4.87E-05
TTK	ENSG00000112742.9	7.18	0.43	2.516	2.40E-17
CDKN3	ENSG00000100526.19	21.296	2.999	2.479	1.88E-12
TACC3	ENSG00000013810.18	45.005	7.355	2.461	2.03E-22
KPNA2	ENSG00000182481.8	100.883	17.7	2.446	5.87E-16
STMN1	ENSG00000117632.20	274.83	49.834	2.44	7.07E-14
WDR72	ENSG00000166415.14	7.975	0.654	2.44	2.17E-05
S100A14	ENSG00000189334.8	183.583	33.412	2.423	8.97E-05
SAPCD2	ENSG00000186193.8	8.965	0.875	2.41	3.81E-16
RP11-6N17.6	ENSG00000264019.1	6.265	0.372	2.404	7.71E-09
ETV4	ENSG00000175832.12	16.26	2.265	2.402	1.73E-10
EXO1	ENSG00000174371.16	6.34	0.395	2.396	9.42E-18
SLC39A4	ENSG00000147804.9	86.509	15.663	2.393	9.52E-26
PRSS8	ENSG00000052344.15	45.215	7.804	2.392	3.76E-10
GTSE1	ENSG00000075218.18	7.705	0.66	2.391	4.51E-19
RAD51	ENSG00000051180.16	10.99	1.304	2.38	4.02E-21
MAD2L1	ENSG00000164109.13	20.619	3.194	2.366	1.68E-12
SPC24	ENSG00000161888.11	11.385	1.41	2.362	2.42E-18
PBK	ENSG00000168078.9	6.945	0.555	2.353	4.08E-13
C4orf48	ENSG00000243449.6	19.425	3.02	2.345	5.32E-16
ORC6	ENSG00000091651.8	10.885	1.345	2.341	1.72E-19
ASPM	ENSG00000066279.16	5.955	0.375	2.339	1.13E-14
SKA3	ENSG00000165480.15	5.88	0.364	2.335	5.94E-19
PRR11	ENSG00000068489.12	9.02	1	2.325	8.69E-13
BUB1B	ENSG00000156970.12	8.94	0.996	2.316	8.54E-18
GINS1	ENSG00000101003.9	8.39	0.908	2.299	3.47E-18
C15orf48	ENSG00000166920.10	27.505	4.82	2.292	1.72E-06
HJURP	ENSG00000123485.11	8.695	0.98	2.292	3.81E-21

SYNE4	ENSG00000181392.14	13.36	1.96	2.278	2.45E-06
GINS2	ENSG00000131153.8	9.79	1.24	2.268	4.20E-17
ULBP2	ENSG00000131015.4	5.204	0.289	2.267	4.15E-11
KIF11	ENSG00000138160.5	8.995	1.085	2.261	1.10E-14
AC110615.1	ENSG00000279909.1	4.475	0.145	2.258	5.40E-05
BUB1	ENSG00000169679.14	11.13	1.536	2.258	1.32E-16
MDK	ENSG00000110492.15	440.713	91.392	2.257	1.68E-07
KLC3	ENSG00000104892.16	5.85	0.434	2.256	6.38E-08
CHI3L1	ENSG00000133048.12	15.985	2.56	2.254	8.67E-03
SFN	ENSG00000175793.11	434.224	91.167	2.239	7.80E-09
SNORD3A	ENSG00000263934.4	233.745	48.881	2.235	3.95E-04
CTSV	ENSG00000136943.10	5.235	0.329	2.23	1.35E-05
MMP9	ENSG00000100985.7	9.08	1.15	2.229	2.50E-03
SKA1	ENSG00000154839.9	4.955	0.285	2.213	6.46E-16
APOBEC3B	ENSG00000179750.15	14.385	2.325	2.21	1.06E-06
IFI6	ENSG00000126709.14	222.543	47.505	2.204	3.08E-04
OIP5	ENSG00000104147.8	5.74	0.465	2.202	1.17E-15
UBE2SP2	ENSG00000224126.2	15.525	2.599	2.199	6.72E-19
HMMR	ENSG00000072571.19	7.46	0.845	2.197	2.35E-13
MCM2	ENSG00000073111.13	26.325	4.972	2.194	1.76E-12
MEX3A	ENSG00000254726.2	5.535	0.435	2.187	4.16E-10
CLIC3	ENSG00000169583.12	20.25	3.685	2.181	1.30E-03
E2F1	ENSG00000101412.12	12.22	1.925	2.176	1.90E-10
LMNB1	ENSG00000113368.11	28.169	5.458	2.175	2.21E-13
SQLE	ENSG00000104549.11	36.255	7.325	2.162	8.89E-13
ECT2	ENSG00000114346.13	16.6	2.958	2.153	1.84E-11
C16orf59	ENSG00000162062.14	6.95	0.79	2.151	2.72E-25
DUXAP9	ENSG00000225210.9	4.945	0.34	2.15	1.92E-08
TYMS	ENSG00000176890.15	50.801	10.669	2.15	6.11E-11
ESM1	ENSG00000164283.12	4.075	0.145	2.148	5.87E-14
CENPU	ENSG00000151725.11	16.374	2.935	2.143	1.47E-15
SHCBP1	ENSG00000171241.8	6.175	0.625	2.143	5.99E-13
CKS1B	ENSG00000173207.12	140.784	31.177	2.14	8.40E-13
DEPDC1B	ENSG00000035499.12	5.145	0.395	2.139	9.20E-15
LIPG	ENSG00000101670.11	5.56	0.499	2.13	5.39E-05
PAFAH1B3	ENSG00000079462.7	94.375	20.837	2.127	4.33E-27
KCNMB2-AS1	ENSG00000237978.5	5.32	0.468	2.106	1.21E-07
CKAP2L	ENSG00000169607.12	4.44	0.265	2.105	1.50E-11
ALDH3B2	ENSG00000132746.14	19.983	3.895	2.1	1.58E-03
SNORA76C	ENSG00000277887.1	3.285	0	2.099	1.14E-03
AC006262.5	ENSG00000268621.5	3.425	0.039	2.09	2.89E-04
CENPK	ENSG00000123219.12	7.3	0.95	2.09	8.61E-13
RP11-498C9.13	ENSG00000263585.1	3.47	0.05	2.09	1.41E-04
POC1A	ENSG00000164087.7	11.19	1.865	2.089	1.46E-15

RP11-386G11.10	ENSG00000258017.1	241.67	56.645	2.074	8.91E-13
HIST1H2BH	ENSG00000275713.1	4.605	0.333	2.072	3.16E-08
ESPL1	ENSG00000135476.11	5.53	0.557	2.068	4.24E-10
VGLL1	ENSG00000102243.12	76.32	17.572	2.058	3.13E-04
RAD51AP1	ENSG00000111247.14	8.075	1.195	2.048	1.42E-09
RP11-867G23.4	ENSG00000254452.1	24.73	5.257	2.04	3.32E-08
PAQR4	ENSG00000162073.13	12.374	2.29	2.023	3.10E-14
NME1	ENSG00000239672.7	174.338	42.215	2.021	5.23E-32
MYEOV	ENSG00000172927.7	4.13	0.27	2.014	2.59E-03
HES2	ENSG00000069812.11	3.8	0.19	2.012	1.51E-03
COL7A1	ENSG00000114270.15	58.854	13.894	2.007	5.58E-07
CYP27A1	ENSG00000135929.8	4.755	22.023	-2	3.49E-06
SCN1B	ENSG00000105711.10	1.97	10.884	-2	2.04E-10
C1R	ENSG00000159403.15	91.979	371.058	-2.001	2.52E-06
NRP2	ENSG00000118257.16	5.715	25.88	-2.001	1.45E-07
LRRK2	ENSG00000188906.13	0.35	4.414	-2.004	4.07E-31
SLC9A9	ENSG00000181804.14	2.065	11.319	-2.007	6.75E-20
LRIG1	ENSG00000144749.13	3.14	15.679	-2.01	2.39E-06
PEG3	ENSG00000198300.12	0.125	3.53	-2.01	1.27E-20
NGFR	ENSG00000064300.8	0.8	6.264	-2.013	6.61E-07
BVES	ENSG00000112276.13	0.445	4.855	-2.019	1.01E-18
AXIN2	ENSG00000168646.12	1.375	8.631	-2.02	3.53E-06
OLFML1	ENSG00000183801.7	1.325	8.434	-2.021	6.31E-17
HSPA2	ENSG00000126803.9	5.295	24.59	-2.023	5.20E-13
PDE5A	ENSG00000138735.15	2.31	12.449	-2.023	3.77E-21
PDE1A	ENSG00000115252.18	0.61	5.55	-2.024	2.97E-25
TMEM220	ENSG00000187824.8	0.87	6.605	-2.024	1.54E-22
CACNB2	ENSG00000165995.18	0.58	5.437	-2.027	9.10E-25
C4B	ENSG00000224389.8	17.63	74.989	-2.028	7.06E-05
ASPA	ENSG00000108381.10	0.15	3.694	-2.029	2.47E-35
CCDC80	ENSG00000091986.15	16.405	70.033	-2.029	2.34E-07
DENND2A	ENSG00000146966.12	1.615	9.675	-2.029	9.25E-15
ADAMTS15	ENSG00000166106.3	0.43	4.845	-2.031	7.57E-10
IFFO1	ENSG00000010295.19	2.495	13.279	-2.031	7.44E-11
APOLD1	ENSG00000178878.12	4.345	20.922	-2.036	2.01E-18
CSPG4	ENSG00000173546.7	3.13	15.965	-2.038	4.27E-08
NFATC4	ENSG00000100968.13	17.255	73.981	-2.038	5.79E-08
PLSCR4	ENSG00000114698.14	2.27	12.429	-2.038	8.67E-14
PPP2R2B	ENSG00000156475.18	2.295	12.537	-2.038	1.08E-03
ADCY2	ENSG00000078295.15	0.195	3.915	-2.04	4.94E-19
ADRB3	ENSG00000188778.4	0.04	3.28	-2.041	4.41E-37
TCEAL1	ENSG00000172465.13	17.115	73.536	-2.041	8.39E-16
S1PR1	ENSG00000170989.8	3.535	17.754	-2.048	2.15E-14

SLC22A3	ENSG00000146477.5	0.43	4.917	-2.049	4.61E-10
HEPH	ENSG00000089472.16	2.02	11.517	-2.051	3.02E-10
RHOB	ENSG00000143878.9	84.176	352.394	-2.053	2.85E-19
AKAP2	ENSG00000241978.9	5.235	24.908	-2.055	2.14E-10
FRY	ENSG00000073910.19	7.655	34.999	-2.056	2.11E-08
AF131217.1	ENSG00000232855.6	0.05	3.368	-2.057	2.46E-47
RHOJ	ENSG00000126785.12	1.77	10.53	-2.057	4.41E-18
TSC22D3	ENSG00000157514.16	47.72	201.725	-2.057	3.05E-13
MT1A	ENSG00000205362.10	0.86	6.738	-2.057	2.42E-11
MICU3	ENSG00000155970.11	0.18	3.915	-2.058	1.68E-43
NCALD	ENSG00000104490.17	1.96	11.335	-2.059	1.06E-15
GARNL3	ENSG00000136895.18	2.19	12.315	-2.061	6.51E-14
TACC1	ENSG00000147526.19	13.79	60.808	-2.063	8.27E-10
SOX17	ENSG00000164736.5	0.75	6.314	-2.063	1.94E-30
NR2F1-AS1	ENSG00000237187.8	2.815	14.952	-2.064	4.30E-08
MEF2C	ENSG00000081189.13	2.15	12.19	-2.066	2.58E-17
NR4A2	ENSG00000153234.13	3.3	17.009	-2.066	6.83E-18
ADHFE1	ENSG00000147576.15	1.46	9.338	-2.071	2.37E-12
PCP4L1	ENSG00000248485.1	5.64	26.945	-2.073	9.71E-03
MYZAP	ENSG00000263155.5	7.5	34.768	-2.073	5.21E-16
CDKL1	ENSG00000100490.9	1.17	8.134	-2.074	1.25E-22
MIR497HG	ENSG00000267532.3	0.89	6.96	-2.074	3.84E-23
BDKRB2	ENSG00000168398.6	3.39	17.508	-2.076	2.70E-15
KLF4	ENSG00000136826.14	14.51	64.471	-2.078	3.68E-15
MCAM	ENSG00000076706.14	24.75	107.892	-2.08	1.36E-12
IL6	ENSG00000136244.11	3	15.914	-2.08	9.30E-10
EDNRA	ENSG00000151617.15	1.91	11.359	-2.086	1.81E-11
CCDC60	ENSG00000183273.6	0.2	4.105	-2.089	3.30E-08
HACD1	ENSG00000165996.13	4.095	20.692	-2.09	1.36E-07
ZFPM2	ENSG00000169946.13	0.48	5.32	-2.094	1.23E-21
LHFP	ENSG00000183722.7	8.495	39.537	-2.094	9.60E-11
FAIM2	ENSG00000135472.8	0.24	4.304	-2.097	7.04E-26
SVIL	ENSG00000197321.14	14.4	64.886	-2.097	3.31E-16
ATF3	ENSG00000162772.16	31.194	136.873	-2.098	2.01E-10
FOXP2	ENSG00000128573.22	0.4	4.998	-2.099	2.49E-24
ANGPTL2	ENSG00000136859.9	10.115	46.639	-2.1	1.40E-06
TPTEP1	ENSG00000100181.21	0.7	6.309	-2.104	2.47E-06
FILIP1L	ENSG00000168386.18	7.52	35.679	-2.106	9.20E-15
TMEM252	ENSG00000181778.4	0.02	3.403	-2.11	2.50E-46
CORO6	ENSG00000167549.18	0.99	7.603	-2.112	9.75E-08
STEAP4	ENSG00000127954.12	1.26	8.767	-2.112	2.63E-09
PKIG	ENSG00000168734.13	30.3	134.265	-2.112	4.29E-16
SGCE	ENSG00000127990.15	8.215	38.928	-2.115	5.43E-08
CAV1	ENSG00000105974.11	30.768	136.745	-2.116	5.20E-07

TSPAN18	ENSG00000157570.11	2.155	12.684	-2.117	1.84E-16
GALNT15	ENSG00000131386.17	0.445	5.295	-2.123	9.09E-22
KLHL13	ENSG00000003096.13	0.785	6.793	-2.126	1.76E-16
KIAA0408	ENSG00000189367.14	0.32	4.764	-2.126	2.60E-29
RBMS3	ENSG00000144642.20	0.54	5.734	-2.129	3.77E-21
CD69	ENSG00000110848.8	2.125	12.685	-2.131	1.38E-06
CTC-296K1.3	ENSG00000267505.1	0.08	3.734	-2.132	6.51E-36
MXRA7	ENSG00000182534.13	37.175	166.745	-2.136	8.37E-16
PCDH18	ENSG00000189184.11	1.385	9.486	-2.136	8.26E-14
GFRA1	ENSG00000151892.14	0.1	3.843	-2.138	4.95E-35
CSRNP1	ENSG00000144655.14	11.375	53.548	-2.14	4.44E-21
NBEA	ENSG00000172915.18	0.515	5.683	-2.141	6.71E-21
AC053503.12	ENSG00000280323.1	0.01	3.458	-2.142	9.33E-34
PRDM8	ENSG00000152784.15	0.725	6.62	-2.143	2.34E-16
DPYSL3	ENSG00000113657.12	13.475	62.983	-2.144	9.60E-09
EPHX2	ENSG00000120915.13	4.64	23.929	-2.144	3.04E-12
ZNF667-AS1	ENSG00000166770.10	5.03	25.714	-2.147	2.77E-05
MITF	ENSG00000187098.14	1.205	8.768	-2.147	3.96E-16
VIPR2	ENSG00000106018.13	0.09	3.836	-2.149	1.07E-52
UTRN	ENSG00000152818.18	3.69	19.866	-2.153	7.31E-12
AXL	ENSG00000167601.11	5.365	27.357	-2.155	5.74E-07
LDB3	ENSG00000122367.19	0.2	4.355	-2.158	1.48E-33
SERPINF1	ENSG00000132386.10	49.755	225.595	-2.158	3.83E-06
CCND2	ENSG00000118971.7	3.615	19.613	-2.159	1.91E-10
RPS6KA2	ENSG00000071242.11	4.05	21.552	-2.159	8.04E-16
NID1	ENSG00000116962.14	11.634	55.452	-2.16	1.52E-04
EPHA3	ENSG00000044524.10	0.585	6.087	-2.161	2.40E-23
CREB5	ENSG00000146592.16	0.635	6.324	-2.163	3.13E-22
THRA	ENSG00000126351.12	7.405	36.662	-2.164	1.02E-15
CLDN11	ENSG00000013297.10	1.615	10.782	-2.172	3.16E-05
SVEP1	ENSG00000165124.17	0.57	6.103	-2.178	1.45E-35
COL21A1	ENSG00000124749.16	0.525	5.919	-2.182	9.23E-23
EPB41L2	ENSG00000079819.16	6.98	35.221	-2.182	1.17E-10
RBPMS	ENSG00000157110.15	57.573	264.854	-2.182	2.79E-11
PLIN4	ENSG00000167676.4	3.03	17.319	-2.184	3.65E-14
RASD1	ENSG00000108551.4	2.135	13.252	-2.185	8.75E-10
FAM110B	ENSG00000169122.11	0.65	6.525	-2.189	2.23E-16
DAAM2	ENSG00000146122.16	1.075	8.471	-2.19	2.68E-16
GHR	ENSG00000112964.13	0.22	4.565	-2.19	4.74E-27
LIFR	ENSG00000113594.9	1.065	8.425	-2.19	7.18E-19
NRG2	ENSG00000158458.19	0.28	4.85	-2.192	6.10E-13
EDNRB	ENSG00000136160.14	1.56	10.705	-2.193	6.58E-16
MEIS1	ENSG00000143995.19	6.84	34.839	-2.193	1.14E-17
OMD	ENSG00000127083.7	0.09	4.005	-2.199	1.84E-22

STARD9	ENSG00000159433.11	0.475	5.773	-2.199	6.59E-32
BMP4	ENSG00000125378.15	3.765	20.933	-2.203	3.42E-04
C3	ENSG00000125730.16	123.677	573.344	-2.204	1.73E-06
PDZD4	ENSG00000067840.12	0.36	5.28	-2.207	4.83E-23
CYTL1	ENSG00000170891.10	0.68	6.77	-2.209	3.06E-15
IGF1	ENSG00000017427.15	0.575	6.282	-2.209	1.05E-09
MAP1A	ENSG00000166963.12	0.775	7.228	-2.213	9.24E-21
TSPAN2	ENSG00000134198.9	2.6	15.704	-2.214	6.30E-12
GAS1	ENSG00000180447.6	2.12	13.48	-2.214	3.27E-05
LMCD1	ENSG00000071282.11	16.845	82.241	-2.222	2.14E-08
CD34	ENSG00000174059.16	11.38	56.828	-2.224	2.35E-19
SNHG14	ENSG00000224078.12	5.545	29.754	-2.232	7.34E-09
TIMP2	ENSG00000035862.12	47.05	225.713	-2.238	9.69E-06
SELENBP1	ENSG00000143416.20	14.36	71.495	-2.239	1.78E-06
SFRP2	ENSG00000145423.4	17.6	86.776	-2.239	1.09E-04
FGF2	ENSG00000138685.12	0.33	5.29	-2.242	2.18E-28
SELM	ENSG00000198832.10	60.675	291.227	-2.244	1.24E-08
COL6A1	ENSG00000142156.14	83.372	399.139	-2.246	6.67E-05
ROR2	ENSG00000169071.14	2.035	13.412	-2.248	9.00E-10
GULP1	ENSG00000144366.15	1.825	12.426	-2.249	4.30E-12
CX3CL1	ENSG00000006210.6	5.38	29.371	-2.251	1.60E-07
HSPB2-C11orf52	ENSG00000254445.1	0.53	6.29	-2.252	7.04E-22
EVA1C	ENSG00000166979.12	8.98	46.618	-2.254	3.50E-10
PTH1R	ENSG00000160801.13	0.605	6.67	-2.257	2.45E-23
EML1	ENSG00000066629.16	2.435	15.442	-2.259	1.80E-13
C15orf59	ENSG00000205363.5	0.845	7.846	-2.261	1.66E-13
SLC24A3	ENSG00000185052.11	1.525	11.1	-2.261	1.10E-16
EBF1	ENSG00000164330.16	0.69	7.104	-2.262	1.69E-27
NEURL1	ENSG00000107954.10	0.31	5.283	-2.262	1.86E-13
GRASP	ENSG00000161835.10	3.815	22.11	-2.263	2.93E-16
RGN	ENSG00000130988.12	0.43	5.867	-2.264	1.93E-16
RGMA	ENSG00000182175.13	0.82	7.745	-2.265	6.74E-08
DUSP2	ENSG00000158050.4	11	56.73	-2.266	3.62E-03
FLNA	ENSG00000196924.14	373.107	1806.147	-2.272	3.18E-12
GPIHBP1	ENSG00000277494.1	0.31	5.332	-2.273	5.68E-31
SPRY1	ENSG00000164056.10	12.685	65.483	-2.28	7.00E-17
PLCD4	ENSG00000115556.13	0.54	6.49	-2.282	8.04E-39
GNAO1	ENSG00000087258.13	0.39	5.775	-2.285	5.89E-23
TSHZ3	ENSG00000121297.6	1.24	9.925	-2.286	5.68E-15
TUB	ENSG00000166402.8	0.44	6.024	-2.286	3.37E-20
MASP1	ENSG00000127241.16	0.17	4.714	-2.288	1.99E-35
HAAO	ENSG00000162882.14	3.465	20.818	-2.289	4.46E-19
ZEB2	ENSG00000169554.16	3.155	19.346	-2.292	3.72E-15
INMT	ENSG00000241644.2	1.035	8.97	-2.293	2.13E-18

BTG2	ENSG00000159388.5	86.9	431.413	-2.298	2.08E-09
CCL21	ENSG00000137077.7	3.75	22.379	-2.299	2.28E-04
ALDH2	ENSG00000111275.12	23.475	119.518	-2.3	8.06E-11
NR3C2	ENSG00000151623.14	0.34	5.6	-2.3	1.43E-29
CTGF	ENSG00000118523.5	35.226	177.486	-2.301	9.01E-11
ATP8B2	ENSG00000143515.16	3.17	19.584	-2.303	8.29E-10
PDE1C	ENSG00000154678.16	0.17	4.777	-2.304	6.00E-37
FZD7	ENSG00000155760.2	4.875	28.051	-2.306	4.15E-15
LRFN5	ENSG00000165379.13	0.1	4.45	-2.309	6.59E-32
NFIX	ENSG00000008441.16	8.06	44.012	-2.313	6.87E-16
WFDC1	ENSG00000103175.10	6.205	34.835	-2.314	4.88E-16
ADAMTSL4	ENSG00000143382.13	7.175	39.682	-2.315	1.39E-05
DCHS1	ENSG00000166341.7	1.205	9.968	-2.315	2.93E-17
PRKCB	ENSG00000166501.12	0.69	7.429	-2.318	1.10E-20
STARD13	ENSG00000133121.20	1.66	12.274	-2.319	1.00E-19
ITPR1	ENSG00000150995.17	3.34	20.667	-2.32	6.89E-22
KCNMA1	ENSG00000156113.20	1.87	13.368	-2.324	8.00E-17
NOVA1	ENSG00000139910.19	0.23	5.179	-2.329	5.17E-25
RP11-887P2.5	ENSG00000258274.1	0.02	4.13	-2.33	1.59E-49
TSPYL2	ENSG00000184205.14	10.26	55.746	-2.333	9.98E-23
SEPP1	ENSG00000250722.5	29.389	152.339	-2.335	1.27E-07
ALDH1A1	ENSG00000165092.12	4.225	25.385	-2.336	1.35E-05
PTGFR	ENSG00000122420.9	0.18	4.965	-2.338	2.78E-32
ABCA6	ENSG00000154262.12	0.945	8.843	-2.339	1.98E-06
MIR4697HG	ENSG00000280237.1	0.115	4.648	-2.341	6.52E-33
P2RY14	ENSG00000174944.8	0.375	5.975	-2.343	4.60E-36
ANXA6	ENSG00000197043.13	29.675	155.007	-2.346	4.90E-11
MEIS2	ENSG00000134138.19	6.105	35.209	-2.349	8.37E-15
RGS5	ENSG00000143248.12	13.919	75.058	-2.35	2.08E-14
DNAJB5	ENSG00000137094.14	3.675	22.85	-2.351	4.84E-18
GAS7	ENSG00000007237.18	1.55	12.025	-2.353	1.26E-15
ST8SIA1	ENSG00000111728.10	0.74	7.889	-2.353	1.34E-14
CTSG	ENSG00000100448.3	0.555	6.957	-2.355	2.81E-14
FAM149A	ENSG00000109794.13	2.005	14.382	-2.356	1.21E-11
TMTC1	ENSG00000133687.15	0.85	8.514	-2.363	4.82E-12
FKBP5	ENSG00000096060.14	9.729	54.306	-2.366	2.54E-08
NPR1	ENSG00000169418.9	1.375	11.29	-2.371	3.26E-16
C1S	ENSG00000182326.14	70.889	371.057	-2.372	3.64E-06
LPP	ENSG00000145012.12	6.69	38.848	-2.373	5.23E-22
FBXL22	ENSG00000197361.6	0.76	8.142	-2.377	2.20E-26
PRICKLE2	ENSG00000163637.11	1.355	11.296	-2.384	5.70E-27
FILIP1	ENSG00000118407.14	0.35	6.085	-2.392	6.66E-35
EPHA7	ENSG00000135333.13	0.225	5.436	-2.393	2.09E-25
HSPB2	ENSG00000170276.5	0.82	8.575	-2.395	1.30E-20

LCN6	ENSG00000267206.5	0.11	4.837	-2.395	3.67E-33
GNG7	ENSG00000176533.12	0.49	6.844	-2.396	1.58E-35
RGAG4	ENSG00000242732.4	0.61	7.488	-2.398	6.84E-19
RRAD	ENSG00000166592.11	3.05	20.357	-2.399	7.93E-06
ZDHHC11B	ENSG00000206077.10	0.295	5.836	-2.4	6.06E-17
MYADM	ENSG00000179820.15	19.575	107.675	-2.401	7.46E-13
RERG	ENSG00000134533.6	1.205	10.658	-2.402	3.36E-16
ADAMTSL3	ENSG00000156218.12	0.115	4.937	-2.413	7.18E-49
APBB1	ENSG00000166313.18	4.34	27.436	-2.413	7.00E-14
TGFB1I1	ENSG00000140682.18	14.14	79.807	-2.416	2.17E-15
pk	ENSG00000091436.16	6.97	41.664	-2.42	6.83E-18
ID4	ENSG00000172201.10	4.67	29.442	-2.425	5.83E-07
GAS6	ENSG00000183087.14	23.835	133.255	-2.434	2.05E-08
RP11-56G10.2	ENSG00000257386.1	0	4.405	-2.434	4.56E-03
ARMCX1	ENSG00000126947.11	2.635	18.684	-2.437	6.16E-12
PDGFRA	ENSG00000134853.11	3.585	23.858	-2.439	4.50E-13
RP11-64B16.2	ENSG00000213144.2	0.92	9.427	-2.441	1.11E-21
SOBP	ENSG00000112320.11	0.48	7.074	-2.448	1.02E-32
TPSD1	ENSG00000095917.13	0.14	5.232	-2.451	5.73E-19
NR2F1	ENSG00000175745.11	4.255	27.789	-2.454	1.64E-13
SLC8A1	ENSG00000183023.18	0.58	7.726	-2.465	5.08E-21
RP11-867G23.10	ENSG00000254510.1	0.15	5.354	-2.466	1.07E-45
FGF10	ENSG00000070193.4	0.05	4.814	-2.469	2.02E-43
LYNX1	ENSG00000180155.18	3.34	23.084	-2.472	2.06E-03
IGSF10	ENSG00000152580.8	0.13	5.28	-2.474	1.66E-28
PELI2	ENSG00000139946.9	0.825	9.208	-2.484	8.24E-25
EGR3	ENSG00000179388.8	1.045	10.447	-2.485	5.83E-21
TCEAL7	ENSG00000182916.7	0.885	9.558	-2.486	6.02E-23
PALM	ENSG00000099864.17	2.87	20.792	-2.493	1.97E-07
PCDH7	ENSG00000169851.15	2.075	16.307	-2.493	1.50E-08
RASL11A	ENSG00000122035.6	3.55	24.678	-2.497	2.67E-15
FERMT2	ENSG00000073712.13	7.145	45.092	-2.501	5.70E-18
MBNL1-AS1	ENSG00000229619.3	0.76	8.961	-2.501	5.29E-27
GSN	ENSG00000148180.16	157.062	898.079	-2.508	1.32E-20
ITGA5	ENSG00000161638.10	31.83	185.827	-2.509	5.90E-12
BIN1	ENSG00000136717.14	11.325	69.317	-2.512	7.91E-13
CSRP1	ENSG00000159176.13	110.744	636.738	-2.513	5.84E-25
GNAL	ENSG00000141404.15	0.83	9.443	-2.513	1.92E-28
F13A1	ENSG00000124491.15	2.585	19.497	-2.515	1.95E-10
C8orf88	ENSG00000253250.2	0.885	9.864	-2.527	9.57E-14
F3	ENSG00000117525.13	11.165	69.276	-2.53	1.63E-04
NXPH3	ENSG00000182575.7	0.72	8.937	-2.53	2.55E-21
RIC3	ENSG00000166405.14	0.37	6.936	-2.534	1.25E-18
RERGL	ENSG00000111404.6	0.19	5.914	-2.539	3.93E-44

PRKAR2B	ENSG00000005249.12	1.595	14.117	-2.542	4.07E-16
LRRN4CL	ENSG00000177363.4	0.58	8.223	-2.545	7.26E-29
TNS2	ENSG00000111077.17	10.275	64.906	-2.547	4.56E-24
TMEM35	ENSG00000126950.7	0.23	6.196	-2.548	4.11E-19
CPXM2	ENSG00000121898.12	1.885	15.92	-2.552	5.01E-12
MGLL	ENSG00000074416.13	6.42	42.545	-2.553	1.44E-15
PMP22	ENSG00000109099.13	23.574	143.444	-2.555	1.57E-11
XPNPEP2	ENSG00000122121.10	0.07	5.325	-2.563	1.06E-32
FBLN2	ENSG00000163520.13	8.48	55.228	-2.568	2.90E-09
CHRDL2	ENSG00000054938.15	1.665	14.874	-2.574	4.74E-04
RBP4	ENSG00000138207.12	0.85	10.034	-2.576	7.81E-05
CPEB1	ENSG00000214575.9	0.16	5.92	-2.577	9.36E-38
GLP2R	ENSG00000065325.12	0.05	5.268	-2.578	8.74E-56
TNFAIP8L3	ENSG00000183578.5	1.085	11.467	-2.58	5.59E-22
VSTM4	ENSG00000165633.12	1.02	11.165	-2.59	2.11E-25
FAM189A2	ENSG00000135063.17	1.01	11.111	-2.591	9.02E-25
COL6A2	ENSG00000142173.14	101.539	617.309	-2.592	1.63E-06
ANK2	ENSG00000145362.16	0.515	8.155	-2.595	7.59E-27
TMEM100	ENSG00000166292.11	0.295	6.83	-2.596	1.62E-27
BCHE	ENSG00000114200.9	0.32	7.001	-2.6	1.01E-17
C20orf166-AS1	ENSG00000174403.15	0.04	5.315	-2.602	1.13E-42
FBXL7	ENSG00000183580.9	1.13	11.964	-2.606	6.16E-24
PDZRN4	ENSG00000165966.14	0.07	5.532	-2.61	6.29E-35
CSDC2	ENSG00000172346.14	0.575	8.634	-2.613	4.00E-13
MFAP5	ENSG00000197614.10	2.535	20.703	-2.618	8.93E-09
LTBP4	ENSG00000090006.17	35.1	220.876	-2.62	2.83E-18
LGI4	ENSG00000153902.13	1.12	12.04	-2.621	4.77E-33
LAMC3	ENSG00000050555.17	3.375	25.96	-2.623	3.66E-13
RP11-903H12.5	ENSG00000259171.1	2.73	22.047	-2.627	1.64E-17
PITX2	ENSG00000164093.15	2.62	21.481	-2.635	1.43E-11
CPED1	ENSG00000106034.17	0.87	10.623	-2.636	3.33E-29
SDC2	ENSG00000169439.11	13.79	91.075	-2.638	4.81E-08
CCDC69	ENSG00000198624.12	8.235	56.976	-2.65	2.07E-25
COLEC12	ENSG00000158270.11	0.91	10.99	-2.65	3.16E-13
MMRN1	ENSG00000138722.9	0.27	6.979	-2.651	2.41E-25
POPDC2	ENSG00000121577.13	1.6	15.339	-2.652	2.86E-20
OSR1	ENSG00000143867.6	6.125	44.009	-2.659	1.25E-06
CCL15-CCL14	ENSG00000275688.4	0.92	11.144	-2.661	7.80E-04
KCND3	ENSG00000171385.9	0.33	7.42	-2.662	3.59E-30
PHYHD1	ENSG00000175287.18	3.015	24.479	-2.666	2.52E-11
SYNE1	ENSG00000131018.22	4.395	33.283	-2.668	1.74E-21
PRDM6	ENSG00000061455.10	0.475	8.388	-2.67	3.60E-31
CLU	ENSG00000120885.19	263.561	1687.825	-2.674	6.70E-08
SBSPON	ENSG00000164764.10	0.855	10.917	-2.683	1.33E-17

ACOX2	ENSG00000168306.12	1.07	12.331	-2.687	4.58E-24
KIAA1644	ENSG00000138944.7	0.17	6.534	-2.687	1.41E-33
OLFML3	ENSG00000116774.11	8.034	57.245	-2.689	1.88E-10
PARM1	ENSG00000169116.11	4.379	33.738	-2.691	7.13E-13
PTGDS	ENSG00000107317.11	20.15	135.832	-2.694	3.06E-08
PLCB4	ENSG00000101333.16	0.58	9.275	-2.701	7.85E-22
TPPP	ENSG00000171368.11	0.4	8.117	-2.703	2.78E-36
CPA3	ENSG00000163751.3	1.86	17.663	-2.706	9.22E-12
STXBP6	ENSG00000168952.15	0.56	9.25	-2.716	1.42E-08
JAM2	ENSG00000154721.14	1.39	14.842	-2.729	3.65E-28
TGFBR3	ENSG00000069702.10	4.995	38.794	-2.731	8.97E-16
PRIMA1	ENSG00000175785.12	0.12	6.435	-2.731	1.09E-39
FOS	ENSG00000170345.9	138.217	925.657	-2.735	6.42E-13
CLDN5	ENSG00000184113.9	5.88	44.849	-2.736	3.77E-16
JPH2	ENSG00000149596.6	1.03	12.531	-2.737	9.46E-18
ARHGEF25	ENSG00000240771.6	6.65	50.13	-2.741	1.97E-15
TPM1	ENSG00000140416.19	105.163	711.702	-2.747	1.25E-18
CKB	ENSG00000166165.12	70.531	480.552	-2.751	4.83E-13
NNAT	ENSG00000053438.8	0.81	11.214	-2.754	1.36E-06
PER1	ENSG00000179094.13	13.355	95.862	-2.754	1.58E-21
COX7A1	ENSG00000161281.10	16.195	115.587	-2.761	9.89E-19
GATA6	ENSG00000141448.8	1.13	13.57	-2.774	1.68E-26
AFF3	ENSG00000144218.18	0.12	6.677	-2.777	9.71E-37
GATA5	ENSG00000130700.6	0.07	6.362	-2.783	3.66E-31
COL4A6	ENSG00000197565.15	3.76	31.78	-2.784	2.73E-11
PPP1R1B	ENSG00000131771.13	2.125	20.669	-2.794	5.25E-04
ZCCHC24	ENSG00000165424.6	2.92	26.195	-2.794	1.30E-21
CSGALNACT1	ENSG00000147408.14	2.22	21.366	-2.796	7.23E-27
RNASE4	ENSG00000258818.3	6.2	49.021	-2.796	1.91E-20
RP11-498E2.9	ENSG00000269970.1	0	5.948	-2.797	9.01E-26
CELF2	ENSG00000048740.17	1.2	14.304	-2.798	1.57E-23
REEP1	ENSG00000068615.16	0.32	8.186	-2.799	3.56E-23
CBX7	ENSG00000100307.12	6.47	51.101	-2.802	3.59E-29
CXCL12	ENSG00000107562.16	5.56	44.749	-2.802	1.80E-13
SLIT2	ENSG00000145147.19	0.52	9.608	-2.803	2.20E-26
EMILIN1	ENSG00000138080.13	16.725	123.021	-2.807	2.00E-07
A2M	ENSG00000175899.14	71.629	509.627	-2.814	3.70E-16
RAMP1	ENSG00000132329.10	7.695	60.225	-2.816	2.10E-12
MT1M	ENSG00000205364.3	1.09	13.75	-2.819	4.66E-10
ZEB1	ENSG00000148516.21	2.23	21.817	-2.82	8.23E-22
WBSCR17	ENSG00000185274.11	0.255	7.884	-2.823	3.17E-30
C16orf45	ENSG00000166780.10	5.29	43.825	-2.833	9.04E-15
ASB2	ENSG00000100628.11	1.075	13.82	-2.836	6.00E-28
FGL2	ENSG00000127951.5	2.345	22.907	-2.837	2.70E-23

AKAP12	ENSG00000131016.16	3.305	29.774	-2.838	1.07E-15
ZFP36	ENSG00000128016.5	127.707	919.304	-2.838	2.76E-18
ATOH8	ENSG00000168874.12	2.275	22.559	-2.847	4.02E-06
TWIST2	ENSG00000233608.3	0.85	12.35	-2.851	4.29E-16
TSPAN7	ENSG00000156298.12	3.05	28.34	-2.857	2.08E-11
DIXDC1	ENSG00000150764.13	1.55	17.482	-2.858	1.77E-27
PHYHIP	ENSG00000168490.13	0.555	10.274	-2.858	1.09E-29
CNRIP1	ENSG00000119865.8	2.275	22.795	-2.861	1.42E-19
PALLD	ENSG00000129116.17	28.665	215.299	-2.866	7.15E-18
PYGM	ENSG00000068976.13	0.33	8.71	-2.868	4.36E-44
CYBRD1	ENSG00000071967.11	8.515	68.977	-2.879	1.25E-13
C1QTNF7	ENSG00000163145.12	0.27	8.369	-2.883	2.63E-52
CFL2	ENSG00000165410.14	7.065	58.509	-2.883	3.38E-21
EGR1	ENSG00000120738.7	42.535	320.117	-2.883	4.93E-13
TACR2	ENSG00000075073.14	0.425	9.509	-2.883	1.85E-25
MGP	ENSG00000111341.9	96.867	721.521	-2.884	2.97E-08
SPEG	ENSG00000072195.14	3.625	33.159	-2.885	4.26E-14
TMOD1	ENSG00000136842.13	1.82	19.93	-2.892	1.05E-25
AQP1	ENSG00000240583.10	25.64	197.096	-2.895	3.18E-21
ANTXR2	ENSG00000163297.16	11.01	88.62	-2.9	2.19E-16
IGFBP6	ENSG00000167779.7	25.114	195.054	-2.908	1.11E-08
ARHGAP6	ENSG00000047648.21	0.68	11.633	-2.911	1.13E-17
LAMA2	ENSG00000196569.11	1.49	17.8	-2.916	1.35E-23
KCNH2	ENSG00000055118.14	0.48	10.178	-2.917	1.91E-19
NEXN	ENSG00000162614.18	2.705	26.983	-2.917	5.37E-22
PLP1	ENSG00000123560.13	0.07	7.085	-2.918	7.39E-63
CYR61	ENSG00000142871.15	46.165	355.611	-2.919	5.75E-14
CLIP3	ENSG00000105270.14	2.735	27.389	-2.926	3.42E-19
MEG3	ENSG00000214548.14	4.505	40.824	-2.926	4.45E-09
KLF9	ENSG00000119138.4	4.155	38.195	-2.927	1.36E-23
GPRASP1	ENSG00000198932.12	0.64	11.49	-2.929	1.07E-39
PPAP2B	ENSG00000162407.8	13.465	109.235	-2.93	1.91E-23
CHRM2	ENSG00000181072.11	0.035	6.901	-2.932	3.24E-39
AGTR1	ENSG00000144891.17	0.205	8.317	-2.951	7.74E-34
GSTM5	ENSG00000134201.10	1.295	16.825	-2.957	3.06E-11
CRYM	ENSG00000103316.10	0.725	12.408	-2.958	1.49E-14
C5orf66-AS1	ENSG00000249082.2	0.37	9.67	-2.961	3.79E-33
SMTN	ENSG00000183963.18	31.474	252.574	-2.965	1.04E-26
SLC2A4	ENSG00000181856.14	0.22	8.602	-2.976	1.68E-41
LINC00865	ENSG00000232229.5	1.765	20.774	-2.977	5.15E-21
FAXDC2	ENSG00000170271.10	1.545	19.053	-2.978	9.48E-33
PAMR1	ENSG00000149090.11	2.26	24.899	-2.99	3.33E-25
GEM	ENSG00000164949.7	4.19	40.36	-2.994	1.83E-20
KLF2	ENSG00000127528.5	5.245	48.858	-2.997	1.15E-27

GYPC	ENSG00000136732.14	8.41	74.125	-2.997	3.04E-14
DMD	ENSG00000198947.14	0.95	14.655	-3.005	6.94E-22
STON1	ENSG00000243244.5	1.72	20.837	-3.005	9.21E-24
CALD1	ENSG00000122786.19	39.53	325.892	-3.012	1.67E-13
TCF21	ENSG00000118526.6	0.725	12.928	-3.013	4.31E-44
ELN	ENSG00000049540.16	5.965	55.308	-3.015	6.43E-11
SLIT3	ENSG00000184347.14	3.4	34.724	-3.021	1.16E-20
SERPINA3	ENSG00000196136.16	1.05	15.668	-3.023	6.58E-08
DACT3	ENSG00000197380.10	1.14	16.406	-3.024	1.21E-22
FGF7	ENSG00000140285.9	1.815	21.94	-3.027	4.54E-15
ITGA7	ENSG00000135424.15	4.08	40.626	-3.035	9.06E-18
ADGRA2	ENSG00000020181.17	4.1	40.84	-3.036	3.02E-16
CRISPLD2	ENSG00000103196.11	8.25	74.975	-3.038	4.22E-15
MAGI2-AS3	ENSG00000234456.7	1.78	22.138	-3.057	5.98E-19
MATN2	ENSG00000132561.13	9.545	86.991	-3.061	9.25E-14
AKR1B10	ENSG00000198074.9	2.785	30.637	-3.063	1.93E-03
KANK2	ENSG00000197256.10	19.745	172.396	-3.063	2.91E-22
ITM2A	ENSG00000078596.10	3.215	34.354	-3.068	1.81E-20
CTC-296K1.4	ENSG00000267405.1	0.1	8.247	-3.071	5.25E-37
MAP1B	ENSG00000131711.14	1.42	19.368	-3.073	4.89E-22
WISP2	ENSG00000064205.10	1.135	16.979	-3.074	1.06E-12
JAM3	ENSG00000166086.12	3.51	37.022	-3.076	1.15E-21
RBM24	ENSG00000112183.14	0.19	9.061	-3.08	1.84E-40
NR4A3	ENSG00000119508.17	0.85	14.671	-3.082	1.38E-23
NEGR1	ENSG00000172260.13	0.32	10.215	-3.087	2.23E-37
DIO3OS	ENSG00000258498.6	0.765	14.044	-3.091	3.17E-21
ITGA8	ENSG00000077943.7	0.74	13.827	-3.091	9.22E-41
TPM2	ENSG00000198467.13	111.816	963.061	-3.095	1.83E-13
DDR2	ENSG00000162733.16	1.55	20.862	-3.1	2.89E-19
RASL12	ENSG00000103710.10	1.965	24.725	-3.117	9.22E-24
IGFBP5	ENSG00000115461.4	22.485	202.79	-3.117	1.02E-09
FAM129A	ENSG00000135842.16	4.55	47.335	-3.122	5.57E-20
IL33	ENSG00000137033.11	2.125	26.263	-3.125	4.82E-10
BOC	ENSG00000144857.14	2.1	26.438	-3.146	3.68E-18
ASB5	ENSG00000164122.8	0.02	8.039	-3.148	1.68E-30
PDE2A	ENSG00000186642.15	1.535	21.465	-3.148	3.41E-31
RASGRP2	ENSG00000068831.18	1.97	25.476	-3.156	3.29E-19
HSD17B6	ENSG00000025423.11	2.345	28.865	-3.158	1.92E-19
SFRP1	ENSG00000104332.11	0.715	14.315	-3.159	2.80E-14
AC002398.12	ENSG00000267328.1	0.485	12.323	-3.165	3.63E-28
OLFM1	ENSG00000130558.18	0.97	16.665	-3.165	2.46E-21
COL14A1	ENSG00000187955.11	4.815	51.33	-3.17	2.84E-18
TPSB2	ENSG00000197253.13	9.095	90.252	-3.176	3.78E-12
ADAMTS9-AS1	ENSG00000241158.5	0.12	9.147	-3.18	9.70E-49

TPSAB1	ENSG00000172236.16	9.294	92.323	-3.18	4.17E-12
ABCA8	ENSG00000141338.13	0.32	10.982	-3.182	1.57E-36
F10	ENSG00000126218.11	0.485	12.583	-3.193	2.24E-48
SOD3	ENSG00000109610.5	11.13	110.068	-3.195	5.05E-11
ZBTB16	ENSG00000109906.13	0.23	10.667	-3.246	2.56E-37
EFEMP1	ENSG00000115380.18	7.9	83.536	-3.248	4.61E-07
RBPMS2	ENSG00000166831.8	2.24	29.965	-3.257	2.27E-21
PDZRN3	ENSG00000121440.14	2.11	28.924	-3.266	1.10E-20
ACACB	ENSG00000076555.15	1.51	23.227	-3.271	1.08E-32
MAOB	ENSG00000069535.13	1.26	20.883	-3.275	1.79E-19
PLAC9	ENSG00000189129.13	2.995	37.871	-3.282	6.92E-25
HLF	ENSG00000108924.13	0.16	10.354	-3.291	1.81E-64
FBLN5	ENSG00000140092.14	7.33	82.414	-3.324	1.11E-18
MSRB3	ENSG00000174099.10	3.095	40.04	-3.325	2.91E-19
KCNMB1	ENSG00000145936.8	0.68	15.864	-3.327	1.58E-28
TCEAL2	ENSG00000184905.8	0.205	11.184	-3.338	2.27E-25
RGS2	ENSG00000116741.7	21.04	222.767	-3.344	1.53E-19
FGFR1	ENSG00000077782.19	6.34	73.753	-3.348	1.74E-14
FAM46B	ENSG00000158246.7	1.415	23.642	-3.351	3.13E-17
CRTAC1	ENSG00000095713.13	0.655	15.903	-3.352	5.31E-03
GPX3	ENSG00000211445.11	26.404	280.828	-3.362	4.81E-12
C11orf96	ENSG00000187479.5	15.18	165.496	-3.363	4.74E-18
RCAN2	ENSG00000172348.14	2.3	33.552	-3.388	8.80E-32
MRVI1	ENSG00000072952.18	2.28	33.434	-3.392	9.59E-24
PID1	ENSG00000153823.18	0.785	17.832	-3.399	3.12E-34
BHMT2	ENSG00000132840.9	0.305	12.776	-3.4	3.75E-45
PPP1R12B	ENSG00000077157.20	5.015	63.375	-3.42	7.54E-29
DUSP1	ENSG00000120129.5	69.896	760.47	-3.425	2.73E-18
AOX1	ENSG00000138356.13	0.305	13.038	-3.427	2.31E-37
LYVE1	ENSG00000133800.8	0.635	16.631	-3.431	2.43E-27
MYOC	ENSG00000034971.14	0.03	10.138	-3.435	6.82E-57
RAI2	ENSG00000131831.17	1.685	28.125	-3.439	1.94E-29
LIMS2	ENSG00000072163.18	8.54	103.766	-3.457	7.56E-24
RP11-166D19.1	ENSG00000255248.6	1.59	27.552	-3.463	1.54E-23
PRAC1	ENSG00000159182.4	0	10.075	-3.469	5.42E-16
PSD	ENSG00000059915.16	1.895	31.3	-3.48	8.11E-26
MYOCD	ENSG00000141052.17	0.165	12.018	-3.482	2.94E-43
SERPINA3	ENSG00000273259.2	2.07	33.31	-3.482	1.10E-07
HSPB8	ENSG00000152137.6	14.615	174.811	-3.493	2.82E-15
ABI3BP	ENSG00000154175.16	2.005	32.953	-3.498	1.97E-24
CADM3	ENSG00000162706.12	0.105	11.514	-3.501	8.60E-31
DTNA	ENSG00000134769.21	1.33	25.437	-3.504	2.24E-22
CILP	ENSG00000138615.5	0.295	13.799	-3.515	2.52E-15
VIT	ENSG00000205221.12	0.03	10.841	-3.523	3.93E-44

CNTN1	ENSG00000018236.14	0.525	16.539	-3.524	1.30E-17
AOC3	ENSG00000131471.6	6.04	80.234	-3.528	3.49E-23
ACKR1	ENSG00000213088.9	3.635	53.288	-3.55	8.91E-17
CLCA4	ENSG00000016602.9	1.425	27.402	-3.55	8.36E-03
PODN	ENSG00000174348.13	5.39	73.868	-3.55	7.45E-13
AP000892.6	ENSG00000280143.1	3.37	50.678	-3.564	3.66E-20
PPP1R14A	ENSG00000167641.10	8.07	106.635	-3.569	3.41E-21
CACNA1H	ENSG00000196557.10	1.955	34.262	-3.577	2.69E-25
ANGPTL1	ENSG00000116194.12	0.21	13.816	-3.614	2.08E-26
MRGPRF	ENSG00000172935.8	2.795	45.468	-3.614	9.93E-22
SHH	ENSG00000164690.7	0.38	15.97	-3.62	1.69E-03
PDLIM3	ENSG00000154553.13	10.18	137.851	-3.635	7.62E-14
SMOC2	ENSG00000112562.18	4.485	67.174	-3.636	2.65E-17
TAGLN	ENSG00000149591.16	216.158	2748.266	-3.662	1.54E-13
MUSTN1	ENSG00000272573.5	1.25	27.505	-3.663	1.38E-28
OGN	ENSG00000106809.10	0.14	13.47	-3.666	1.42E-33
PKDCC	ENSG00000162878.12	2.03	37.542	-3.669	3.25E-14
PLN	ENSG00000198523.5	1.02	24.689	-3.669	2.32E-19
ADAMTS1	ENSG00000154734.14	7.21	104.672	-3.686	3.47E-27
APOD	ENSG00000189058.8	9.33	132.422	-3.691	2.01E-08
HPSE2	ENSG00000172987.12	0.14	13.848	-3.703	3.80E-25
ITIH5	ENSG00000123243.14	2.645	46.658	-3.709	1.75E-30
PRUNE2	ENSG00000106772.17	0.825	23.325	-3.736	3.57E-25
SPON1	ENSG00000262655.3	2.385	44.195	-3.739	2.21E-16
HAND2-AS1	ENSG00000237125.8	0.18	14.838	-3.747	6.84E-38
CES1	ENSG00000198848.12	5.175	82.335	-3.755	1.66E-09
ADCY5	ENSG00000173175.14	0.585	20.451	-3.758	1.44E-31
ACTA2	ENSG00000107796.12	138.942	1908.005	-3.77	2.17E-16
SDPR	ENSG00000168497.4	1.64	35.07	-3.772	2.30E-33
CRYAB	ENSG00000109846.7	12.56	185.324	-3.78	2.63E-19
FOSB	ENSG00000125740.13	13.27	195.293	-3.782	6.74E-13
LMO3	ENSG00000048540.14	0.715	22.971	-3.805	1.03E-23
SRPX	ENSG00000101955.14	4.445	75.285	-3.808	1.51E-18
FXYD6	ENSG00000137726.15	5.475	90.803	-3.826	1.50E-17
ATP1A2	ENSG00000018625.14	0.12	15.182	-3.853	6.82E-48
CHRDL1	ENSG00000101938.14	0.15	15.812	-3.87	2.94E-27
ACTA2-AS1	ENSG00000180139.11	0.45	20.36	-3.881	1.73E-29
MIR143HG	ENSG00000249669.7	0.4	19.681	-3.885	1.53E-42
PLA2G2A	ENSG00000188257.10	14.554	232.27	-3.907	2.49E-06
MYL9	ENSG00000101335.9	115.76	1758.28	-3.913	7.50E-17
FOXF1	ENSG00000103241.6	2.025	44.84	-3.922	1.64E-31
DCN	ENSG00000011465.16	81.433	1251.939	-3.926	5.18E-12
LINC01082	ENSG00000269186.1	0.98	29.104	-3.926	2.90E-38
C16orf89	ENSG00000153446.15	0.51	23.079	-3.995	2.24E-41

NR4A1	ENSG00000123358.19	27.059	447.921	-4	1.56E-21
FABP4	ENSG00000170323.8	10.09	176.791	-4.003	1.59E-04
NCAM1	ENSG00000149294.16	0.3	20.054	-4.017	2.04E-31
SGCA	ENSG00000108823.15	0.945	30.645	-4.024	8.03E-25
SPARCL1	ENSG00000152583.12	26.215	444.704	-4.034	1.61E-25
SORBS2	ENSG00000154556.17	3.93	83.912	-4.106	1.52E-24
ADAM33	ENSG00000149451.17	0.725	28.715	-4.107	1.99E-35
HIF3A	ENSG00000124440.15	0.25	21.109	-4.145	1.87E-33
RBFOX3	ENSG00000167281.18	0.06	17.938	-4.159	8.35E-43
ADGRD1	ENSG00000111452.12	0.45	25.042	-4.167	9.92E-35
MAMDC2	ENSG00000165072.9	0.42	24.536	-4.169	7.20E-34
PTGIS	ENSG00000124212.5	1.61	46.163	-4.176	4.52E-17
PTGS1	ENSG00000095303.14	3.52	80.949	-4.18	1.01E-19
MFAP4	ENSG00000166482.11	16.469	318.819	-4.194	1.63E-16
TNS1	ENSG00000079308.16	5.69	122.515	-4.207	1.17E-25
HAND2	ENSG00000164107.8	0.64	30.054	-4.243	7.94E-26
PCOLCE2	ENSG00000163710.7	1.53	49.545	-4.32	2.78E-24
CLEC3B	ENSG00000163815.5	3.67	92.702	-4.327	2.67E-40
TNXB	ENSG00000168477.17	2.27	65.992	-4.357	7.40E-31
MYLK	ENSG00000065534.18	10.945	246.977	-4.376	2.41E-21
FXYD1	ENSG00000266964.5	1.78	57.375	-4.392	9.15E-35
RP11-394O4.5	ENSG00000269936.3	1.25	47.41	-4.427	2.71E-25
FAM107A	ENSG00000168309.16	0.62	34.484	-4.453	1.60E-51
DPT	ENSG00000143196.4	1.18	49.038	-4.521	4.52E-21
PDK4	ENSG00000004799.7	3.055	92.905	-4.533	5.94E-31
HSPB7	ENSG00000173641.17	0.93	44.778	-4.568	1.68E-18
PRELP	ENSG00000188783.5	1.385	55.97	-4.578	8.66E-28
CASQ2	ENSG00000118729.11	0.155	26.838	-4.591	1.92E-26
P2RX1	ENSG00000108405.3	0.435	33.969	-4.607	1.87E-31
SORBS1	ENSG00000095637.20	2.485	84.846	-4.623	8.52E-26
CFD	ENSG00000197766.7	9.755	336.846	-4.973	1.06E-31
FENDRR	ENSG00000268388.5	1.375	73.684	-4.975	3.04E-33
FLNC	ENSG00000128591.15	1.36	73.482	-4.98	4.65E-24
C2orf40	ENSG00000119147.9	0.25	46.439	-5.246	1.42E-40
SYNPO2	ENSG00000172403.10	1.67	102.465	-5.276	6.78E-31
CCL14	ENSG00000276409.4	1.9	111.638	-5.279	1.92E-26
SCARA5	ENSG00000168079.16	0.175	45.04	-5.292	1.81E-64
ACTG2	ENSG00000163017.13	29.733	1316.919	-5.422	4.23E-18
LMOD1	ENSG00000163431.12	2.76	166.784	-5.48	1.25E-26
PI16	ENSG00000164530.13	0.165	51.662	-5.498	1.51E-47
PGM5	ENSG00000154330.12	1.15	105.226	-5.627	1.08E-32
FHL1	ENSG00000022267.16	6.415	367.295	-5.634	7.94E-26
SYNM	ENSG00000182253.14	2.185	159.063	-5.651	4.61E-30
PCP4	ENSG00000183036.10	0.965	112.483	-5.852	8.22E-17

CNN1	ENSG00000130176.7	11.275	739.783	-5.915	1.28E-20
C7	ENSG00000112936.18	0.645	123.426	-6.241	1.71E-28
HSPB6	ENSG00000004776.11	3.43	334.718	-6.244	1.34E-29
ADH1B	ENSG00000196616.12	0.275	107.653	-6.413	1.60E-44
PGM5-AS1	ENSG00000224958.5	0.08	110.268	-6.687	1.41E-41
MYH11	ENSG00000133392.16	9.785	1543.391	-7.162	6.81E-24
ACTC1	ENSG00000159251.6	0.425	228.136	-7.329	4.94E-19
DES	ENSG00000175084.11	9.275	2026.227	-7.624	2.27E-16
