# Supplementary material for: Three Prognostic Biomarkers Correlate with Immune Checkpoint Blockade Response in Bladder Urothelial Carcinoma
Source: Int J Genomics. 2022 May 26;2022:3342666. doi: 10.1155/2022/3342666 (PMC9162857; doi:10.1155/2022/3342666)
Supplement: Supplementary Materials — Supplementary 1: different expression genes from GEPIA database. Supplementary 2: different expression genes from Oncomine database. Supplementary 3: original source data: the relevant code or R software. [file 3342666.f1.zip › Supplemetary 2- oncomine-Diff (1).pdf]

Supplementary 2 Different expression genes from Oncomine database

Gene symbol	Gene symbol	Gene symbol
AADAC	FCER1G	PLCB1
AAK1	FCGR1A	PLCB4
ABCB5	FCGR2A	PLCG1
ABCC5	FCGR2B	PLCXD2
ABCF3	FCGR2C	PLD1
ABCG5	FCGR3A	PLEK
ABCG8	FCGR3B	PLEKHF2
ABHD1	FCRL1	PLEKHH2
ABHD12	FCRL2	PLEKHO1
ABI1	FCRL3	PLGLB1
ABI3BP	FCRL4	PLGLB2
ABRA	FCRL5	PLK1S1
ACAP2	FCRL6	PLTP
ACBD5	FCRLA	PLUNC
ACOT8	FCRLB	PLXDC2
ACP1	FDPS	PMEPA1
ACSS1	FERD3L	PMF1
ACSS2	FERMT1	PMVK
ACTB	FETUB	PNO1
ACTG1	FEZ2	PNPT1
ACTG2	FGF12	POFUT1
ACTL6A	FIGLA	POGK
ACTR2	FILIP1L	POGZ
ACTR5	FITM2	POLE4
ACYP2	FKBP1A	POLR2H
ADA	FKBP1B	POLR2K
ADAM15	FKBP9	POLR3F
ADAM17	FLAD1	POMC
ADAM33	FLG	POP1
ADAMTS4	FLG2	POTEG
ADAMTSL4	FLJ25363	POU2F1
ADAR	FLJ45079	POU6F2
ADCY10	FLRT3	PPFIA1
ADCY3	FN3K	PPIAL4A
ADCYAP1R1	FN3KRP	PPIAL4E
ADD2	FNDC3B	PPM1B
ADI1	FNDC4	PPM1G
ADIG	FOSL2	PPOX
ADIPOQ	FOXA2	PPP1CB
ADNP	FOXK2	PPP1R16B
ADRA1D	FOXN2	PPP1R2

AFMID	FOXS1	PPP1R3D
AFTPH	FSCN1	PPP3R1
AGBL5	FSCN2	PQLC3
AGR3	FSHR	PRCC
AHCY	FXR1	PREB
AHR	FYTTD1	PREPL
AHSA2	FZD6	PREX1
AHSG	GABPB2	PRKCE
AIM2	GABRR3	PRKCI
AIMP2	GAD2	PRND
ALCAM	GALM	PRNP
ALDH9A1	GALNT14	PROCR
ALG3	GATAD2B	PROKR1
ALK	GBA	PROKR2
ALLC	GCKR	PROS1
ALMS1	GCNT7	ProSAPiP1
ALOX5AP	GDAP1L1	PRPF3
AMPH	GDF5	PRPS1L1
AMZ1	GDF7	PRPSAP1
ANAPC11	GEM	PRTFDC1
ANGPT1	GEMIN6	PRUNE
ANGPT4	GEN1	PSMB4
ANKMY2	GET4	PSMD2
ANKRD26	GFPT1	PSMD4
ANKRD30A	GFRA4	PSME4
ANKRD46	GGT7	PSMF1
ANKRD5	GGTLC1	PSMG3
ANKRD53	GHRH	PTCHD3
ANP32E	GHRHR	PTDSS1
ANTXR1	GHSR	PTF1A
ANXA4	GINS1	PTGIS
ANXA9	GKN1	PTPN1
AOAH	GKN2	PTPRA
AP2M1	GLCCI1	PTPRT
APBB1IP	GMCL1	PUM2
APCDD1L	GNA12	PUS10
APCS	GNAS	PVRL3
APEX2	GNB4	PVRL4
APH1A	GNRH2	PXMP4
APLF	GOLPH3L	PYCR1
APOA1BP	GON4L	PYDC2
APOA2	GPA33	PYGB
APOB	GPATCH4	PYGO2
AQP1	GPCPD1	PYHIN1

AQP10	GPN1	R3HDML
ARFGEF2	GPNMB	RAB10
ARHGAP12	GPR113	RAB11FIP5
ARHGAP21	GPR15	RAB13
ARHGAP25	GPR158	RAB18
ARHGAP30	GPR160	RAB1A
ARHGDIA	GPR161	RAB22A
ARHGEF11	GPR75	RAB25
ARHGEF2	GPS1	RAB40B
ARHGEF33	GREB1	RAC1
ARL13B	GRHL1	RAC3
ARL16	GRHL2	RAD21L1
ARL5B	GRID2IP	RAD51AP2
ARL6	GSS	RAD54B
ARL8B	GTF2A1L	RADIL
ARMC3	GTSF1L	RAE1
ARMC4	GUCA1C	RAG1AP1
ARNT	GZF1	RALGAPA2
ARPM1	HAAO	RALGAPB
ASAP2	HADHA	RALY
ASB3	HADHB	RAPGEF5
ASH1L	HAO1	RASGRP3
ASIP	HAPLN2	RASSF2
ASPRV1	HAX1	RBAK
ASPSCR1	HCK	RBBP9
ASXL1	HCN3	RBCK1
ASXL2	HDAC9	RBKS
ATAD2B	HDGF	RBL1
ATF6	HEATR2	RBM12
ATL2	HEATR5B	RBM12B
ATP11B	HEPACAM2	RBM38
ATP1A2	HEXDC	RBM39
ATP1A4	HGS	RBPJL
ATP1B1	HHLA2	RCSD1
ATP5E	HIBADH	RDH14
ATP6V1B1	HIST2H2AB	REG1A
ATP6V1C1	HIST2H2AC	REG1B
ATP6V1C2	HIST2H2BE	REG3A
ATP6V1E2	HK2	REG3G
ATP8B2	HM13	REL
ATP9A	HMGB1	REM1
ATRN	HMGB1L1	RETNLB
AUP1	HNF4A	RFC4
AURKA	HNRNPA2B1	RFNG

AVP	HNRPLL	RFX5
AZI1	HORMAD1	RG9MTD1
AZIN1	HOXA10	RGPD1
B3GALTL	HOXA11	RGPD2
B3GNT2	HOXA13	RGS22
B3GNT5	HOXA3	RGS4
B4GALT3	HOXA7	RGS5
B4GALT5	HOXA9	RHBG
BAALC	HPCAL1	RHOB
BAHCC1	HRG	RHOQ
BAMBI	HRNBP3	RIMS2
BANF2	HRNR	RIMS4
BBS9	HRSP12	RIN2
BBX	HS1BP3	RIPK2
BCAN	HSD17B7	RIT1
BCAS1	HSPA12B	RNASEH1
BCAS4	HSPA6	RNF114
BCL11A	HSPC159	RNF144A
BCL2L1	HSPH1	RNF168
BCL6	HTR3C	RNF19A
BDH1	HTR3D	RNF216
BFSP1	HTR3E	RNF24
BGLAP	HTRA2	ROBLD3
BHLHE40	IAH1	ROCK2
BIRC5	ICA1	ROMO1
BIRC6	ID1	RORC
BLCAP	ID2	RPA3
BMP10	IDH3B	RPL22L1
BMP2	IFI16	RPL24
BMP7	IFT172	RPL35A
BMPER	IFT52	RPL39L
BNIPL	IFT57	RPN2
BOLA1	IGF2BP2	RPRD1B
BOLA3	IGF2BP3	RPRD2
BPI	IGSF11	RPS27
BPIL1	IGSF8	RPS27A
BPIL3	IGSF9	RPS7
BRE	IKZF3	RPTN
BRP44	IL1RAP	RRBP1
BTBD3	IL6	RRM2
BZW2	IL6R	RRM2B
C10orf113	ILDR2	RSAD2
C10orf114	ILF2	RSPH10B
C10orf140	IMPG2	RSPH10B2

C10orf47	INHBA	RSPO2
C10orf67	INO80B	RSPO4
C10orf68	INSM1	RTKN
C13orf26	INSRR	RTN4
C13orf33	INTS1	RTP1
C17orf101	INTS3	RTP2
C17orf55	INTS8	RTP4
C17orf56	IQCE	RUNX1T1
C17orf62	IQCG	RUSC1
C17orf70	IQGAP3	RXFP4
C17orf89	ISG20L2	RXRG
C17orf90	ISM1	S100A1
C1D	ISPD	S100A10
C1orf104	ITCH	S100A11
C1orf110	ITGA10	S100A12
C1orf111	ITGB1	S100A13
C1orf112	ITGB1BP1	S100A14
C1orf115	ITGB8	S100A16
C1orf156	ITLN1	S100A2
C1orf182	ITLN2	S100A3
C1orf189	ITPA	S100A4
C1orf192	ITPR1	S100A5
C1orf204	ITSN2	S100A6
C1orf226	IVL	S100A7
C1orf230	JAG1	S100A7A
C1orf43	JAZF1	S100A7L2
C1orf51	JPH2	S100A8
C1orf54	JTB	S100A9
C1orf56	KATNAL1	SALL4
C1orf61	KBTBD12	SAMD12
C1orf66	KCNB1	SAMD7
C1orf68	KCNF1	SAMHD1
C1orf77	KCNG1	SCAMP3
C1orf85	KCNG3	SCAND1
C1orf92	KCNJ10	SCIN
C1QTNF1	KCNJ9	SCNM1
C20orf103	KCNK12	SCRT2
C20orf106	KCNK15	SDC1
C20orf107	KCNK3	SDC2
C20orf108	KCNMB2	SDC4
C20orf111	KCNMB3	SDCBP2
C20orf112	KCNN3	SDHC
C20orf114	KCNS1	SEC14L1
C20orf117	KCNS2	SEC22B

C20orf118	KCNV1	SEC23B
C20orf12	KDELR2	SEC61A2
C20orf123	KHK	SEC62
C20orf132	KIAA0226	SECTM1
C20orf134	KIAA0406	SEL1L2
C20orf141	KIAA0907	SELE
C20orf144	KIAA1217	SELENBP1
C20orf152	KIAA1429	SELL
C20orf160	KIAA1462	SELP
C20orf165	KIAA1524	SEMA4A
C20orf173	KIAA1755	SEMA4F
C20orf177	KIAA1841	SEMA6C
C20orf185	KIDINS220	SEMG1
C20orf186	KIF16B	SEMG2
C20orf194	KIF3B	SENP2
C20orf196	KIF3C	SENP5
C20orf197	KIF5B	SENP7
C20orf202	KIFAP3	SERINC3
C20orf24	KIR2DL4	SERTAD2
C20orf26	KIRREL	SETDB1
C20orf27	KLF10	SF3B14
C20orf29	KLF11	SF3B4
C20orf3	KLHDC9	SFRS6
C20orf30	KLHL24	SFRS7
C20orf4	KLHL6	SFT2D2
C20orf43	KLHL7	SFXN5
C20orf46	KLRAQ1	SGK2
C20orf54	KLRC2	SH2D1B
C20orf7	KNG1	SH2D2A
C20orf70	KPRP	SH3YL1
C20orf71	KRTCAP2	SHC1
C20orf72	KRTCAP3	SHE
C20orf79	L3MBTL	SI
C20orf85	LAMP3	SIGLEC1
C20orf94	LAPTM4A	SIRPA
C20orf96	LAPTM4B	SIRPB2
C2CD4D	LASS2	SIRPD
C2orf16	LBH	SIRPG
C2orf18	LBP	SIRT7
C2orf3	LBX2	SIX2
C2orf34	LCE1A	SIX3
C2orf39	LCE1B	SKAP2
C2orf42	LCE1C	SKIL
C2orf43	LCE1E	SLA2

C2orf44	LCE1F	SLAMF1
C2orf48	LCE2A	SLAMF6
C2orf50	LCE2B	SLAMF7
C2orf53	LCE2C	SLAMF8
C2orf61	LCE2D	SLAMF9
C2orf63	LCE3A	SLC12A5
C2orf65	LCE3D	SLC13A3
C2orf7	LCE3E	SLC1A4
C2orf70	LCE4A	SLC23A2
C2orf71	LCE5A	SLC24A3
C2orf73	LCE6A	SLC25A10
C2orf74	LCLAT1	SLC25A32
C2orf78	LELP1	SLC25A44
C2orf79	LENEP	SLC26A7
C2orf81	LEPREL1	SLC27A3
C2orf84	LFNG	SLC29A4
C2orf86	LGALS3BP	SLC2A10
C2orf90	LHCGR	SLC2A2
C3orf21	LINGO4	SLC30A6
C3orf26	LIPH	SLC30A8
C3orf34	LMLN	SLC32A1
C3orf43	LMNA	SLC35C2
C3orf59	LMX1A	SLC39A1
C3orf70	LNP1	SLC39A12
C7orf10	LOC283999	SLC3A1
C7orf11	LOC375190	SLC4A11
C7orf16	LOC388946	SLC4A1AP
C7orf26	LOC402644	SLC4A5
C7orf27	LOC642502	SLC5A6
C7orf28A	LOC647309	SLC7A1
C7orf30	LOC728819	SLC7A14
C7orf31	LOR	SLC8A1
C7orf46	LOXL3	SLC9A8
C7orf70	LPIN1	SLMO2
C8orf37	LPIN3	SLPI
C8orf38	LPP	SMC6
C8orf47	LRCH3	SMCP
CA14	LRP12	SMEK2
CACNB2	LRPPRC	SMG5
CADM3	LRRC31	SMOX
CALB1	LRRC33	SMYD5
CALM2	LRRC34	SNAI1
CAMK1D	LRRC45	SNAP25
CAMK2N2	LRRC52	SNAPIN

CANT1	LRRC69	SNPH
CAPN13	LRRIQ4	SNRNP27
CAPN14	LRRN4	SNRPB
CARD11	LRRTM1	SNRPB2
CASQ1	LRRTM4	SNRPG
CASS4	LTBP1	SNTA1
CBFA2T2	LY9	SNTG2
CBLB	LYSMD1	SNX13
CBLN4	LYZL1	SNX17
CBX2	LYZL2	SNX21
CBX3	MACC1	SNX27
CBX4	MACROD2	SNX31
CBX8	MAD1L1	SNX5
CCDC104	MAEL	SOCS3
CCDC121	MAFB	SOCS5
CCDC126	MAFG	SOS1
CCDC129	MAFK	SOSTDC1
CCDC137	MAGEF1	SOX11
CCDC142	MANBAL	SOX12
CCDC19	MAP1LC3A	SP4
CCDC39	MAP3K13	SPAG1
CCDC50	MAP3K8	SPAG4
CCDC54	MAP4K3	SPAG6
CCDC7	MAP6D1	SPAST
CCDC75	MAPRE1	SPATA16
CCDC85A	MAPRE3	SPATA2
CCDC88A	MASP1	SPDYA
CCNE2	MASTL	SPEF1
CCT3	MATN2	SPINLW1
CCT4	MATN3	SPINT3
CCT7	MATN4	SPINT4
CD1A	MAVS	SPO11
CD1B	MBOAT2	SPR
CD1C	MC3R	SPRED2
CD1D	MCCC1	SPRR1A
CD1E	MCEE	SPRR1B
CD207	MCF2L2	SPRR2A
CD244	MCFD2	SPRR2B
CD247	MCL1	SPRR2D
CD40	MCM8	SPRR2E
CD47	MDH1	SPRR2F
CD48	MECOM	SPRR2G
CD5L	MEF2D	SPRR3
CD7	MEIS1	SPRR4

CD84	MEMO1	SPTA1
CD93	MEX3A	SPTBN1
CD96	MFI2	SPTLC3
CDC123	MFN1	SRBD1
CDC25B	MFSD2B	SRC
CDC42SE1	MGLL	SRD5A2
CDCA7L	MGST3	SRXN1
CDH17	MICALL2	SSR2
CDH22	MINA	SST
CDH26	MIOS	SSTR4
CDK13	MKKS	ST3GAL6
CDK5RAP1	MKX	ST6GAL1
CDKL4	MLLT10	STAMBP
CDS2	MLLT11	STAU1
CEBPB	MMD2	STK3
CELF3	MMP24	STK31
CENPA	MMP9	STK35
CENPB	MNDA	STK4
CENPO	MOBKL1B	STON1
CEP170	MOCS3	STON1-GTF2A1L
CEP250	MOGS	STRA13
CEP68	MORC1	STRN
CEP97	MORN2	STS
CFHR3	MPHOSPH10	STX16
CGN	MPP6	STX19
CGREF1	MPP7	SULF2
CHAC2	MPZ	SUMF1
CHD6	MPZL1	SUN1
CHGB	MRPL12	SUN5
CHMP4B	MRPL19	SUPT7L
CHRD	MRPL24	SV2A
CHRNB2	MRPL33	SVIL
CIB4	MRPL47	SYBU
CKS1B	MRPL53	SYCP2
CLCN2	MRPL9	SYNGR2
CLDN1	MRPS21	SYS1
CLDN11	MRPS26	SYT11
CLDN16	MSH2	TACR1
CLDND1	MSH6	TADA1
CLEC4F	MSRB2	TAF1B
CLIP4	MSTO1	TAGLN2
CLK2	MTA3	TARS2
CMPK2	MTDH	TASP1
CNRIP1	MTHFD2	TBC1D20

COL28A1	MTIF2	TBC1D23
COL8A1	MTMR11	TBCCD1
COLEC10	MTPAP	TBCD
COLEC11	MTUS2	TBL1XR1
COMMD1	MTX1	TBX19
COMMD7	MUC1	TCF15
COPA	MUC4	TCF23
COX4I2	MXD1	TCHH
COX6C	MYADML2	TCHHL1
COX7A2L	MYBL2	TCTEX1D2
CPNE1	MYCN	TDRD10
CPOX	MYH15	TDRKH
CPSF3	MYH7B	TET3
CPXM1	MYL9	TEX19
CRABP2	MYLK2	TEX261
CRCT1	MYNN	TFAP2C
CREB3L4	MYO3A	TFG
CREB5	MYT1L	TFRC
CREG1	NAA20	TGFA
CREM	NAALADL2	TGIF2
CRIM1	NAGK	TGM2
CRIPT	NANP	TGM3
CRLS1	NAPB	TGM6
CRNKL1	NARF	TH1L
CRNN	NAT8	THADA
CRP	NAT8B	THBD
CRTC2	NBAS	THBS3
CRYBG3	NBN	THEM4
CRYGS	NBPF10	THEM5
CSE1L	NBPF14	THNSL1
CSNK2A1	NBPF16	THOC4
CSRP2BP	NCALD	THPO
CST1	NCBP2	THSD7A
CST11	NCEH1	THUMPD2
CST2	NCOA1	TIA1
CST3	NCOA3	TIMP2
CST4	NCOA5	TIPRL
CST5	NCOA6	TK1
CST7	NCSTN	TLX2
CST8	NDRG3	TM4SF19
CST9	NDUFB5	TM7SF4
CST9L	NDUFS2	TM9SF4
CSTF1	NEBL	TMC2
CSTL1	NECAB1	TMC6

CTCFL	NECAB3	TMC8
CTHRC1	NES	TMCO1
CTNNA2	NEURL2	TMEM105
CTNNBL1	NFATC2	TMEM17
CTSA	NFE2L3	TMEM178
CTSK	NFKBIZ	TMEM18
CTSS	NFS1	TMEM184A
CTSZ	NHLH1	TMEM189
CYCS	NINL	TMEM189-UBE2V1
CYP1B1	NIPAL2	TMEM196
CYP24A1	NIT1	TMEM207
CYP26B1	NIT2	TMEM214
CYS1	NKX2-2	TMEM41A
CYTH1	NKX2-4	TMEM45A
CYTH3	NLGN1	TMEM55A
DAGLB	NLRC4	TMEM64
DAP3	NME7	TMEM67
DARC	NNAT	TMEM74
DBNDD2	NOL10	TMEM79
DCAF13	NOP56	TMEM90B
DCAF6	NOS1AP	TMOD4
DCAF8	NOTCH2NL	TMX4
DCBLD2	NOTO	TNFAIP8L2
DCST1	NOTUM	TNFSF10
DCST2	NPB	TNIK
DCTN1	NPEPL1	TNK2
DCUN1D1	NPLOC4	TNNC2
DCXR	NPR1	TNRC18
DDR2	NPSR1	TNRC6C
DDRGK1	NPVF	TOMM20
DDX1	NPY	TOMM34
DDX27	NR1I3	TOMM40L
DECR1	NRBP1	TOMM7
DEDD	NRP1	TOMM70A
DEFB115	NRSN2	TOP1
DEFB116	NRXN1	TOX2
DEFB118	NSFL1C	TP53I3
DEFB119	NSUN3	TP53INP1
DEFB121	NSUN6	TP53INP2
DEFB123	NT5C1B	TP53RK
DEFB124	NTRK1	TP53TG5
DEFB125	NTSR2	TP63
DEFB126	NUDCD1	TPM3
DEFB127	NUDT5	TPRG1

DEFB128	NUF2	TPRKB
DEFB129	NUP210L	TPTE
DEFB132	NUPL2	TPX2
DENND4B	NXPH1	TRA2A
DFNA5	NXT1	TRA2B
DGKB	OAZ3	TRAT1
DGKG	ODC1	TRHR
DGUOK	ODF1	TRIB2
DHFRL1	OLFML2B	TRIB3
DHX35	OR10J1	TRIM46
DHX57	OR10J3	TRMT6
DLG1	OR10J5	TRMT61B
DLGAP4	OR10K1	TRPC4AP
DNAH11	OR10K2	TSHZ2
DNAJB11	OR10R2	TSPAN10
DNAJC1	OR10T2	TSPYL5
DNAJC19	OR10X1	TSPYL6
DNAJC27	OR10Z1	TSSC1
DNMT3A	OR13G1	TSTD1
DNMT3B	OR14C36	TTC14
DNTTIP1	OR2AK2	TTC15
DOK1	OR2G2	TTC24
DOK5	OR2G3	TTC27
DPM1	OR2G6	TTC31
DPM3	OR2L8	TTC32
DPPA2	OR2M3	TTC35
DPPA4	OR2M7	TTC7A
DPT	OR2T29	TTLL9
DPY19L1	OR2T33	TTPAL
DPY19L4	OR2T34	TUBB1
DPY30	OR2T35	TUFT1
DPYS	OR2T4	TXNDC3
DPYSL5	OR2T5	UAP1
DQX1	OR2T6	UBAP2L
DSN1	OR4K1	UBE2C
DSTN	OR4K2	UBE2Q1
DTD1	OR4K5	UBE2V1
DTNB	OR4M1	UBL3
DUS1L	OR4N2	UBOX5
DUSP11	OR4Q3	UBQLN4
DUSP12	OR5AC2	UBR5
DUSP15	OR5H1	UBXN2A
DUSP23	OR5H14	UBXN7
DUSP27	OR5H15	UCK2

DVL3	OR5H2	UFC1
DYNC2LI1	OR5H6	UGP2
DYNLRB1	OR5K1	UHMK1
DYSF	OR5K2	UNCX
DYSFIP1	OR5K3	UPF2
DZIP3	OR5K4	UQCC
E2F1	OR6F1	USF1
E2F6	OR6K2	USP13
EBAG9	OR6K3	USP21
EBF4	OR6K6	USP34
ECE2	OR6N1	USP36
ECHDC3	OR6N2	USP42
ECM1	OR6P1	USP6NL
ECT2	OR6Y1	USPL1
EDEM2	OSBPL3	UTS2D
EDN3	OSGIN2	UTS2R
EFCAB8	OSR1	VANGL2
EFEMP1	OSR2	VAPB
EFNA1	OST4	VAX2
EFNA3	OSTalpha	VHLL
EFNA4	OSTN	VIT
EFR3B	OTOF	VPS13B
EGR4	OTOR	VPS16
EHBP1	OTUD1	VPS45
EHD3	OTUD6B	VPS54
EHHADH	OTUD7B	VPS72
EIF2AK1	OTX1	VPS8
EIF2B4	OVOL2	VRK2
EIF2B5	OXER1	VSIG8
EIF2S2	OXR1	VSNL1
EIF3E	OXT	VSTM2L
EIF4A2	P4HB	VSX1
EIF4G1	PABPC1	VWA5B2
EIF5A2	PABPC1L	VWDE
EIF6	PAIP2B	WAC
ELFN1	PAK2	WBP1
ELMO1	PAK7	WDR35
ELMO2	PANK2	WDR43
EMILIN1	PAPOLB	WDR45L
EMILIN3	PAPOLG	WDR53
EML4	PAQR6	WDR54
EML6	PARD3	WDR92
EMX1	PARD6B	WFDC10A
ENGASE	PARL	WFDC10B

ENKUR	PAX1	WFDC11
ENPP7	PBX1	WFDC12
ENSA	PBXIP1	WFDC13
ENTPD6	PCBP1	WFDC2
ENY2	PCGF1	WFDC3
EPAS1	PCIF1	WFDC5
EPB41L1	PCK1	WFDC6
EPC1	PCNA	WFDC8
EPCAM	PCNP	WFDC9
EPHA6	PCP4L1	WIPI2
EPHB3	PCSK2	WISP2
EPT1	PCYOX1	XCL1
ERGIC3	PCYT1A	XCL2
ERLEC1	PCYT2	XDH
ESF1	PDE1C	XKR7
ESRP1	PDE4DIP	XPO1
ETAA1	PDE6G	XRN2
ETV1	PDIA6	YEATS2
ETV3	PDP1	YIPF4
ETV3L	PDRG1	YME1L1
ETV5	PDSS1	YPEL5
EVX1	PDYN	YWHAB
EXOC3L2	PEA15	YWHAQ
EXOC6B	PEAR1	YWHAZ
EYA2	PELI1	YY1AP1
F11R	PEX13	ZBED2
F5	PEX19	ZBP1
FAM110A	PEX5L	ZBTB11
FAM110C	PFDN2	ZBTB7B
FAM113A	PFDN4	ZCCHC3
FAM126A	PFN4	ZDHHC19
FAM131A	PGCP	ZDHHC4
FAM136A	PGLYRP3	ZFP36L2
FAM150B	PGLYRP4	ZFP64
FAM161A	PGS1	ZFPM2
FAM176A	PHACTR3	ZHX3
FAM179A	PHC3	ZMAT3
FAM189B	PHF14	ZMYND8
FAM49A	PHF20	ZNF12
FAM55C	PHLDB2	ZNF133
FAM63A	PI3	ZNF217
FAM65C	PI4KB	ZNF334
FAM83C	PIGF	ZNF335
FAM83D	PIGM	ZNF337

FAM84A	PIGT	ZNF341
FAM92A1	PIGU	ZNF343
FAM98A	PIGX	ZNF438
FANCL	PIGZ	ZNF512
FASN	PIK3CA	ZNF513
FASTKD5	PIP4K2A	ZNF638
FBXL18	PIP5K1A	ZNF639
FBXO11	PKDCC	ZNF687
FBXO41	PKHD1L1	ZNF706
FBXO43	PKIG	ZNF750
FBXO45	PKLR	ZNF831
FBXO48	PLAGL2	ZNF853
FCER1A	PLB1	ZNFX1
ZSWIM1	ZSWIM3	ZPLD1
