# Supplementary material for: Three Prognostic Biomarkers Correlate with Immune Checkpoint Blockade Response in Bladder Urothelial Carcinoma
Source: Int J Genomics. 2022 May 26;2022:3342666. doi: 10.1155/2022/3342666 (PMC9162857; doi:10.1155/2022/3342666)
Supplement: Supplementary Materials — Supplementary 1: different expression genes from GEPIA database. Supplementary 2: different expression genes from Oncomine database. Supplementary 3: original source data: the relevant code or R software. [file 3342666.f1.zip › Supplemetary 3-relevant code or R software.pdf]

1. The associations between the expression of 11 hub genes and OS was evaluated using the R survival package.

```
#install.packages("survival")
setwd("C:\\Users\\86186\\Desktop\\CKS1B")
library(survival)
rt=read.table("survivalInput.txt",header=T,sep="\t")
rt$futime=rt$futime/365
a=rt[,"expression"]<median(rt[,"expression"])
diff=survdiff(Surv(futime, fustat) ~a,data = rt)
pValue=1-pchisq(diff$chisq,df=1)
pValue=round(pValue,5)
fit <- survfit(Surv(futime, fustat) ~ a, data = rt)
summary(fit)
pdf(file="survival.pdf")
plot(fit, lty = 2:3,col=c("red","blue"),xlab="time (day)",ylab="surival rate",
        main=paste("surival curve (p=", pValue ,")",sep=""))
legend("topright", c("CKS1B high expression", "CKS1B low expression"), lty = 2:3,
col=c("red","blue"))
dev.off()
```

2. Risk difference analysis

```
#if (!requireNamespace("BiocManager", quietly = TRUE))
#       install.packages("BiocManager")
#BiocManager::install("limma")



library(limma)
expFile="symbol.txt"
riskFile="risk.all.txt"
logFCfilter=1
fdrFilter=0.01
setwd("C:\\Users\\86186\\Desktop\\BLCA\\25.riskDiff")

rt=read.table(expFile, header=T, sep="\t", check.names=F)
rt=as.matrix(rt)
rownames(rt)=rt[,1]
exp=rt[,2:ncol(rt)]
dimnames=list(rownames(exp), colnames(exp))
data=matrix(as.numeric(as.matrix(exp)), nrow=nrow(exp), dimnames=dimnames)
data=avereps(data)

group=sapply(strsplit(colnames(data),"\\-"), "[", 4)
group=sapply(strsplit(group,""), "[", 1)
group=gsub("2", "1", group)
```

```
data=data[,group==0]
data=t(data)
rownames(data)=gsub("(.*?)\\-(.*?)\\-(.*?)\\-(.*?)\\-.*", "\\1\\-\\2\\-\\3", rownames(data))
data=avereps(data)
data=t(data)

risk=read.table(riskFile, header=T, sep="\t", check.names=F, row.names=1)
sameSample=intersect(colnames(data), row.names(risk))
data=data[,sameSample]
risk=risk[sameSample,]

riskLow=risk[risk$risk=="low",]
riskHigh=risk[risk$risk=="high",]
dataLow=data[,row.names(riskLow)]
dataHigh=data[,row.names(riskHigh)]
data=cbind(dataLow,dataHigh)
data=data[rowMeans(data)>1,]
conNum=ncol(dataLow)
treatNum=ncol(dataHigh)
Type=c(rep(1,conNum), rep(2,treatNum))

outTab=data.frame()
for(i in row.names(data)){
	rt=data.frame(expression=data[i,], Type=Type)
	wilcoxTest=wilcox.test(expression ~ Type, data=rt)
	pvalue=wilcoxTest$p.value
	conGeneMeans=mean(data[i,1:conNum])
	treatGeneMeans=mean(data[i,(conNum+1):ncol(data)])
	logFC=log2(treatGeneMeans)-log2(conGeneMeans)
	conMed=median(data[i,1:conNum])
	treatMed=median(data[i,(conNum+1):ncol(data)])
	diffMed=treatMed-conMed
	if( ((logFC>0) & (diffMed>0)) | ((logFC<0) & (diffMed<0)) ){

	outTab=rbind(outTab,cbind(gene=i,lowMean=conGeneMeans,highMean=treatGeneMeans,l
ogFC=logFC,pValue=pvalue))
	}
}
pValue=outTab[,"pValue"]
fdr=p.adjust(as.numeric(as.vector(pValue)), method="fdr")
outTab=cbind(outTab, fdr=fdr)

outDiff=outTab[(                    abs(as.numeric(as.vector(outTab$logFC)))>logFCfilter                    &
as.numeric(as.vector(outTab$fdr))<fdrFilter),]
```

```
write.table(outDiff, file="riskDiff.txt", sep="\t", row.names=F, quote=F)
```

3.Immune function

```
if (!requireNamespace("BiocManager", quietly = TRUE))
    install.packages("BiocManager")
BiocManager::install("limma")

if (!requireNamespace("BiocManager", quietly = TRUE))
    install.packages("BiocManager")
BiocManager::install("GSVA")

if (!requireNamespace("BiocManager", quietly = TRUE))
      install.packages("BiocManager")
BiocManager::install("GSEABase")

install.packages("pheatmap")
install.packages("reshape2")


library(limma)
library(GSVA)
library(GSEABase)
library(pheatmap)
library(reshape2)

expFile="symbol.txt"
gmtFile="immune.gmt"
riskFile="risk.all.txt"
setwd("C:\\Users\\86186\\Desktop\\27.immFunction")
rt=read.table(expFile, header=T, sep="\t", check.names=F)
rt=as.matrix(rt)
rownames(rt)=rt[,1]
exp=rt[,2:ncol(rt)]
dimnames=list(rownames(exp),colnames(exp))
mat=matrix(as.numeric(as.matrix(exp)),nrow=nrow(exp),dimnames=dimnames)
mat=avereps(mat)
mat=mat[rowMeans(mat)>0,]


geneSet=getGmt(gmtFile, geneIdType=SymbolIdentifier())

#ssgsea
ssgseaScore=gsva(mat, geneSet, method='ssgsea', kcdf='Gaussian', abs.ranking=TRUE)
#定义 ssGSEA score
```

```
normalize=function(x){
	return((x-min(x))/(max(x)-min(x)))}
#对 ssGSEA score
data=normalize(ssgseaScore)
ssgseaOut=rbind(id=colnames(data), data)
write.table(ssgseaOut, file="immFunScore.txt", sep="\t", quote=F, col.names=F)

group=sapply(strsplit(colnames(data),"\\-"), "[", 4)
group=sapply(strsplit(group,""), "[", 1)
group=gsub("2", "1", group)
data=t(data[,group==0])
rownames(data)=gsub("(.*?)\\-(.*?)\\-(.*?)\\-(.*?)\\-.*", "\\1\\-\\2\\-\\3", rownames(data))
data=t(avereps(data))

risk=read.table(riskFile,header=T,sep="\t",row.names=1,check.names=F)
lowSample=row.names(risk[risk$risk=="low",])
highSample=row.names(risk[risk$risk=="high",])
lowData=data[,lowSample]
highData=data[,highSample]
data=cbind(lowData, highData)
conNum=ncol(lowData)
treatNum=ncol(highData)
sampleType=c(rep(1,conNum), rep(2,treatNum))

sigVec=c()
for(i in row.names(data)){
	test=wilcox.test(data[i,] ~ sampleType)
	pvalue=test$p.value
	Sig=ifelse(pvalue<0.001,"***",ifelse(pvalue<0.01,"**",ifelse(pvalue<0.05,"*","")))
	sigVec=c(sigVec, paste0(i, Sig))
}
row.names(data)=sigVec

Type=c(rep("Low risk",conNum), rep("High risk",treatNum))
Type=factor(Type, levels=c("Low risk", "High risk"))
names(Type)=colnames(data)
Type=as.data.frame(Type)
pdf("heatmap.pdf", width=8, height=4.6)
pheatmap(data,
		annotation=Type,
		color = colorRampPalette(c(rep("blue",5), "white", rep("red",5)))(100),
		cluster_cols =F,
		cluster_rows =T,
		scale="row",
```

```
        show_colnames=F,
        show_rownames=T,
        fontsize=7,
        fontsize_row=7,
        fontsize_col=7)
dev.off()
```

4. Maftools

```
#if (!require("BiocManager"))
#       install.packages("BiocManager")
#BiocManager::install("maftools")


library(maftools)
setwd("C:\\Users\\86186\\Desktop\\BLCA\\29.maftools")
risk=read.table("risk.all.txt", header=T, sep="\t", check.names=F)
outTab=risk[,c(1, ncol(risk))]
colnames(outTab)=c("Tumor_Sample_Barcode", "Risk")
write.table(outTab, file="ann.txt", sep="\t", quote=F, row.names=F)

geneNum=20
geneMut=read.table("geneMut.txt", header=T, sep="\t", check.names=F, row.names=1)
gene=row.names(geneMut)[1:geneNum]

ann_colors=list()
col=c("blue", "red")
names(col)=c("low", "high")
ann_colors[["Risk"]]=col

pdf(file="low.pdf", width=6, height=6)
maf=read.maf(maf="low.maf", clinicalData="ann.txt")
oncoplot(maf=maf, clinicalFeatures="Risk", genes=gene, annotationColor=ann_colors,
keepGeneOrder=T)
dev.off()

pdf(file="high.pdf", width=6, height=6)
maf=read.maf(maf="high.maf", clinicalData="ann.txt")
oncoplot(maf=maf, clinicalFeatures="Risk", genes=gene, annotationColor=ann_colors,
keepGeneOrder=T)
dev.off()
```

5. Risk TMB

```
install.packages("ggpubr")

if (!requireNamespace("BiocManager", quietly = TRUE))
    install.packages("BiocManager")
BiocManager::install("limma")


library(limma)
library(ggpubr)
library(ggplot2)
setwd("C:\\Users\\86186\\Desktop\\BLCA\\30.riskTMB")
tmb=read.table("TMB.txt", header=T, sep="\t", check.names=F, row.names=1)


risk=read.table("risk.all.txt", header=T, sep="\t", check.names=F, row.names=1)

sameSample=intersect(row.names(tmb), row.names(risk))
tmb=tmb[sameSample,,drop=F]
risk=risk[sameSample,,drop=F]
data=cbind(tmb, risk)
data$TMB=log2(data$TMB+1)

data$risk=ifelse(data$risk=="high", "High-risk", "Low-risk")
group=levels(factor(data$risk))
data$risk=factor(data$risk, levels=c("Low-risk", "High-risk"))
comp=combn(group,2)
my_comparisons=list()
for(i in 1:ncol(comp)){my_comparisons[[i]]<-comp[,i]}

boxplot=ggviolin(data, x="risk", y="TMB", fill="risk",
                  xlab="",
                  ylab="Tumor tmbation burden (log2)",
                  legend.title="",
                  palette = c("#0066FF","#FF0000"),
                  add = "boxplot", add.params = list(fill="white"))+
    stat_compare_means(comparisons = my_comparisons)

pdf(file="riskTMB.pdf", width=5, height=4.5)
print(boxplot)
dev.off()
```

6. TMB survival

```
install.packages("survival")
install.packages("survminer")
```

```
library(survival)
library(survminer)
tmbFile="TMB.txt"
riskFile="risk.all.txt"
setwd("C:\\Users\\86186\\Desktop\\31.tmbSur")

risk=read.table(riskFile, header=T, sep="\t", check.names=F, row.names=1)
tmb=read.table(tmbFile, header=T, sep="\t", check.names=F, row.names=1)
sameSample=intersect(row.names(tmb), row.names(risk))
tmb=tmb[sameSample,,drop=F]
risk=risk[sameSample,,drop=F]
data=cbind(risk, tmb)

res.cut=surv_cutpoint(data, time = "futime", event = "fustat", variables =c("TMB"))
cutoff=as.numeric(res.cut$cutpoint[1])
tmbType=ifelse(data[,"TMB"]<=cutoff, "L-TMB", "H-TMB")
scoreType=ifelse(data$risk=="low", "low risk", "high risk")
mergeType=paste0(tmbType, "+", scoreType)

bioSurvival=function(surData=null, outFile=null){
	diff=survdiff(Surv(futime, fustat) ~ group, data=surData)
	length=length(levels(factor(surData[,"group"])))
	pValue=1-pchisq(diff$chisq, df=length-1)
	if(pValue<0.001){
		pValue="p<0.001"
	}else{
		pValue=paste0("p=",sprintf("%.03f",pValue))
	}
	fit <- survfit(Surv(futime, fustat) ~ group, data = surData)
	#print(surv_median(fit))

	bioCol=c("#FF0000","#0066FF","#6E568C","#7CC767","#223D6C","#D20A13","#FFD121","#0
88247","#11AA4D")
	bioCol=bioCol[1:length]
	surPlot=ggsurvplot(fit,
	                   data=surData,
	                   conf.int=F,
	                   pval=pValue,
	                   pval.size=6,
	                   legend.title="",
	                   legend.labs=levels(factor(surData[,"group"])),
	                   font.legend=10,
```

```
                                          legend = c(0.8, 0.8),
                                          xlab="Time(years)",
                                          break.time.by = 1,
                                          palette = bioCol,
                                          surv.median.line = "hv",
                                          risk.table=F,
                                          cumevents=F,
                                          risk.table.height=.25)
        pdf(file=outFile, onefile = FALSE, width=5.5, height=4.8)
        print(surPlot)
        dev.off()
}

data$group=tmbType
bioSurvival(surData=data, outFile="TMB.survival.pdf")

data$group=mergeType
bioSurvival(surData=data, outFile="TMB-risk.survival.pdf")
```
